# Exploration Deficits Under Ecological Conditions as a Marker of Apathy in Frontotemporal Dementia

**DOI:** 10.3389/fneur.2019.00941

**Published:** 2019-08-28

**Authors:** Bénédicte Batrancourt, Karen Lecouturier, Johan Ferrand-Verdejo, Vincent Guillemot, Carole Azuar, David Bendetowicz, Raffaella Migliaccio, Armelle Rametti-Lacroux, Bruno Dubois, Richard Levy

**Affiliations:** ^1^Inserm U 1127, CNRS UMR 7225, Sorbonne Universités, UPMC Univ Paris 06, Institut du Cerveau et de la Moelle épiniére (ICM), FRONTlab, Paris, France; ^2^Institut Pasteur, Centre de Bioinformatique, Biostatistique et Biologie Intégrative (C3BI), Paris, France; ^3^AP-HP, Groupe Hospitalier Pitiè-Salpêtrière, Département de Neurologie, Institut de la Mèmoire et de la Maladie d'Alzheimer (IM2A), Paris, France; ^4^AP-HP, Groupe Hospitalier Pitiè-Salpêtrière, Département de Neurologie, Behavioral Neuropsychiatry Unit, Paris, France

**Keywords:** apathy, disinhibition, motivation, frontotemporal lobar degeneration, behavior, neurodegenerative diseases, dementia, prefrontal cortex

## Abstract

Apathy is one of the six clinical criteria for the behavioral variant of frontotemporal dementia (bvFTD), and it is almost universal in this disease. Although its consequences in everyday life are debilitating, its underlying mechanisms are poorly known, its assessment is biased by subjectivity and its care management is very limited. In this context, we have developed “*ECOCAPTURE*,” a method aimed at providing quantifiable and objective signature(s) of apathy in order to assess it and identify its precise underlying mechanisms. *ECOCAPTURE* consists of the observation and recording of the patient's behavior when the participant is being submitted to a multiple-phase scenario reproducing a brief real-life situation. It is performed in a functional exploration platform transformed into a fully furnished waiting room equipped with a video and sensor-based data acquisition system. This multimodal method allowed video-based behavior analyses according to predefined behavioral categories (exploration behavior, sustained activities or inactivity) and actigraphy analyses from a 3D accelerometer. The data obtained were also correlated with behavioral/cognitive tests and scales assessing global cognitive efficiency, apathy, cognitive disinhibition, frontal syndrome, depression and anxiety. Here, bvFTD patients (*n* = 14) were compared to healthy participants (*n* = 14) during the very first minutes of the scenario, when the participants discovered the room and were encouraged to explore it. We showed that, in the context of facing a new environment, healthy participants first explored it and then engaged in sustained activities. By contrast, bvFTD patients were mostly inactive and eventually explored this new place, but in a more irregular and less efficient mode than normal subjects. This exploration deficit was correlated with apathy, disinhibition and cognitive and behavioral dysexecutive syndromes. These findings led us to discuss the presumed underlying mechanisms responsible for the exploration deficit (an inability to self-initiate actions, to integrate reward valuation and to inhibit involuntary behavior). Altogether, these results pave the way for simple and objective assessment of behavioral changes that represents a critical step for the evaluation of disease progression and efficacy of treatment in bvFTD.

## Introduction

Apathy can be defined as a quantitative reduction in voluntary or goal-directed behavior (GDB) (1, 2). It is the most frequent behavioral syndrome in neurological and psychiatric diseases, such as Alzheimer's disease (AD), Parkinson's disease, stroke and schizophrenia (3–10). Apathy is usually assessed by questionnaires (scales) administered to the patient and/or caregiver and providing information about the patient's internal state, thoughts and past activities, globally suggesting a loss of motivation to perform daily activities (11, 12). However, these scales are biased by the subjective nature of the patient or caregiver's perspective (13). As fever can be suspected from thirst or chills but properly evaluated only by measuring temperature using an objective tool (a thermometer), we asked ourselves the following question by analogy: is possible for a clinician to obtain a measure of apathy from direct patient observation with the help of a simple and objective tool? For this purpose, we have developed a research project called “*ECOCAPTURE*” with the aim of obtaining a quantifiable composite signature of apathy. It is an evaluation program to assess apathy in patients and healthy subjects by objective quantitative measures that are collected in an ecological setting. *ECOCAPTURE* consists of the observation and multimodal recording and analyses of the subject's behavior when performing a multiple-phase scenario. This scenario reproduces a brief, real-life situation. One phase of the ECOCAPTURE scenario consists of discovering a new environment (waiting room) and being encouraged to explore it. This real-life situation is relevant to the study of apathy because it favors the generation of GDB. The clinical observation of exploration behavior can be transformed into quantifiable measures and may help us objectively assess apathy and elucidate its underlying pathological mechanisms.

Frontotemporal dementia (FTD) is the second most common form of neurodegenerative dementia after AD and is a good model in which to study apathy for several reasons. First, the presence of apathy is a major criterion for the clinical diagnosis of the behavioral variant of FTD (bvFTD) (14). It is almost universal in bvFTD (15) and responsible for major disabilities in everyday life (14, 16). Second, as anosognosia and loss of insight are highly frequent in bvFTD patients, it is likely that apathy would be misestimated if evaluated using currently available apathy scales. Third, neurodegeneration in bvFTD mostly affects the frontal lobes, which are considered crucial for generating and controlling GDB (17). Fourth, the underlying mechanisms of apathy are poorly known in general (18) and in bvFTD in particular (19).

Within this framework, we hypothesize that apathy in bvFTD arises from at least three pathological underlying mechanisms altering the generation and control of GDB: defects in valuation, cognitive elaboration and self-initiation (1, 13). Each of these mechanisms possibly relies on distinct fronto-subcortical loops (circuits) not equally affected in bvFTD, in which neurodegeneration mainly affects brain networks involved in affective and initiation processing rather than in cognitive aspects (19–23). As a consequence, the study of patients with bvFTD will provide valuable information to better understand the pathological mechanisms underlying apathy.

The general aims of this study were as follows: (i) to provide simple, objective, quantifiable markers to evaluate apathy in bvFTD patients, and (ii) to determine the cognitive/psychological mechanisms explaining apathetic behavior in this disease. For those purposes, we conducted a study in bvFTD patients and compared patients to healthy subjects using the *ECOCAPTURE* protocol. Our clinical experience suggests that apathetic FTD patients spontaneously lack initiative in non-automatic situations. Our working hypothesis was that in a situation where participants are encouraged to explore a new environment, we would observe a drastic reduction in GDB in the most apathetic bvFTD patients during the exploration sequence. These behavioral changes should be observed in the scenario of exploring a new place, while several behavioral measures that reflect the presence or absence of voluntary action could be recorded using videos and body sensors.

## Materials and Methods

### Overview

Healthy participants and bvFTD patients were submitted to the *ECOCAPTURE* protocol (clinicaltrials.gov: NCT03272230, NCT02496312), designed to obtain objective and quantifiable signatures of behavioral syndromes such as apathy or disinhibition. The study took place in a functional exploration platform transformed into a fully furnished waiting room equipped with video monitoring and a sensor-based data acquisition system that tracked the participant's behavior. In this room, participants followed a multiple-phase scenario testing their abilities to generate GDB. The scenario was made of successive phases to disambiguate self-generated behavior from externally driven behaviors. It started with a freely moving (self-guided) phase (first phase), when participants entered the room and were encouraged to explore it. This was followed by several different contexts (e.g., an externally guided phase consisting of filling out a questionnaire that required to use different items present in the rooms and in a specific order, or a decision-making context in which participants must adapt to an unexpected situation such as a social interaction). Behavior was monitored by means of multimodal tools (video and a sensor-based acquisition system), then categorized using ethograms and statistically processed. In the present work, we focused on self-initiated behavior, i.e., behavior in the very first minutes of the scenario, during which participants entered the room and were encouraged to freely explore it. In-room behavior was analyzed in isolation and in correlation with selected neuropsychological tests.

This study obtained formal approval from the local ethical research committee.

### Participants

We enrolled 14 patients with bvFTD (8 females and 6 males; mean age: 65.29 ± 11.28) who were included in the study according to the international consensus criteria (14). They were compared to a group of 14 sex- and age-matched healthy controls (HC) (7 females and 7 males; mean age: 62.14 ± 7.46), with the same level of education. The demographic characteristics are described in Table 1. For age, the condition of equality of population variances was verified (Levene's test; Pr [>F] = 0.1306). The two populations did not differ for age (Welch's *t*-test, *p* = 0.3938). The variable “level of education” did not follow a normal distribution. Group comparisons using the Mann-Whitney-Wilcoxon test were therefore applied. The two populations were comparable in gender and education level (*p* = 1).

**Table 1:** Demographic and neuropsychological characteristics of the studied populations.

	**Healthy participants**	**bvFTD patients**	***P*-value (type of test)**
*n*	14	14	
F/M	7/7	8/6	
Age± SD/[range]	62.14 ± 7.46/[46–72]	65.29 ± 11. 28/[45–82]	NS (WTT)
Level of education (level)	7 ± 1.75 [3–7]	7 ± 1.75 [3–7]	NS (WRS)
Years of schooling (yo)	14.28 ± 3.7 [7–17]	14.28 ± 3.7 [7–17]	NS (WTT)
Starkstein Apathy Scale (/42)	6.71 ± 2.76	17 ± 6.39	*p* = 3.16e-05 (WTT)
DAS (/72)	14.64 ± 8.69	18.64 ± 12.33	NS (WTT)
DAS-cognition subscore (/24)	4 ± 3.09	9.93 ± 5.27	*p* = 0.0016 (WTT)
DAS-emotion subscore (/24)	9.36 ± 2.68	8.64 ± 3.37	NS (WTT)
DAS-initiation subscore (/24)	7 ± 3.40	7.93 ± 6.45	NS (WTT)
Hayling B-A (/)	29.14 ± 10.27	81.07 ± 86.92	NS (WRS)
Hayling E (/)	2.14 ± 1.23	26.43 ± 11.37	*p* = 5.21e-06 (WRS)
MMSE (/30)	29.64 ± 0.63	22.93 ± 2.4	*p* = 7.49e-06 (WRS)
FAB (/18)	17.57 ± 0.65	12 ± 3.55	*p* = 4.03e-05 (WRS)
SEA (/30)	26.63 ± 1.11	18.38 ± 3.17	*p* = 8.30e-08 (WRS)
HAD-D (/21)	1.36 ± 1.9	6.14 ± 3.37	*p* = 0.0003 (WRS)
HAD-A (/21)	5.29 ± 1.68	7.36 ± 5.05	NS (WTT)

*n, number; F, female; M, male; yo, years old; DAS, Dimensional Apathy Scale; MMSE, Mini-Mental Status Examination; FAB, Frontal Assessment Battery; SEA, Social & Emotional Assessment; HAD, Hospital Anxiety and Depression (-A, Anxiety; -D, Depression); NS, non-significant; WRS, Wilcoxon rank sum; WTT, Welch's t-test*.

### Multiphase ECOCAPTURE Scenario (Figure 1)

**Figure 1:**
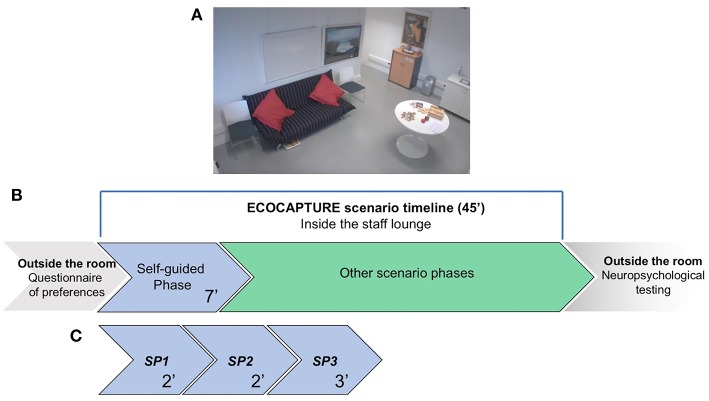
Schematic representation of the ECOCAPTURE scenario in the “waiting room.” **(A)** Screenshot from one of the video cameras installed in the room, showing some of the furniture and objects in the room. **(B)** A global and schematic representation of the full scenario timeline. Before entering the room, participants completed a questionnaire indicating their preferences for music, food, drinks and magazine reading. Then, they entered the room and were submitted to the multiple-step ECOCAPTURE scenario, starting with the self-guided (freely moving) phase, which was followed by several other phases (not discussed in the present study). After the ECOCAPTURE session, they performed classic neuropsychological tests. **(C)** The self-guided (freely moving) phase. It lasted 7 min (called the “*FULL PERIOD*”) and was divided into three “*SUBPERIODS”* (*SP1, SP2*, and *SP3*) of 2, 2, and 3 min. During this phase, participants were encouraged to explore the room and interact with the different items.

The study took place into an experimental platform dedicated to the functional exploration of human behavior (PRISME, ICM core facility, Salpêtrière hospital, Paris, France). The platform was transformed into a fully furnished waiting room (mimicking a staff lounge) equipped with different data acquisition systems that tracked the participant's behavior. This room contained specific objects (refreshments, magazines, games, furniture) that provided opportunities to interact (Supplementary Material-A). In this room, participants were submitted to a 45-min multiple-step scenario (Figure 1). This scenario followed a predetermined order, starting with a freely moving (or self-guided) phase, during which participants entered the room, discovered it and were explicitly encouraged to explore it. This freely moving phase, lasting 7 min, represents the focus of the present study, allowing us to analyze exploration behavior (i.e., how the participant behaves when discovering a novel environment to which she/he should adapt). Consequently, the measures focused on evaluating the subject's capacity to explore the room, initiate behaviors and maintain them to reach specific goals. This first phase was followed by several other phases that are not described here because they are beyond the scope of the presented study (Figure 1).

Prior to the experimental session, we asked participants and/or caregivers to indicate their preferences for food, drinks, magazine reading and music, and we customized the room accordingly (Supplementary Material-B). Participants were told that they would participate in a research project taking place in a room that serves as a staff lounge and that their behavior would be tracked from the very first minutes in the room and recorded by video cameras located in the room.

The first phase was self-guided: subjects were encouraged to make themselves at ease in the room and use the space (e.g., to sit on a chair around a table or on a sofa) and objects (refreshments, magazines, games, etc.) at their own convenience.

### ECOCAPTURE Multimodal Recording With Video and Sensor-Based Acquisition System

**Figure 2:**
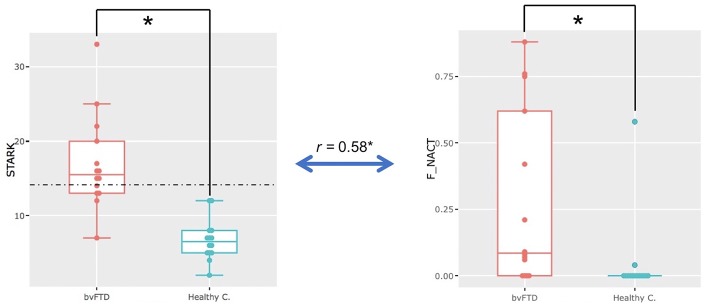
Apathy in bvFTD. Significant difference in Starkstein Apathy Scale scores (STARK) (*p* = 0.00003) and on the *non-activity* metric (F_NACT) (*p* = 0.002) in the whole freely moving (self-guided) phase (i.e., the *FULL PERIOD*) between bvFTD and healthy participants (healthy c.). Significant positive correlation (*r* = 0.58, *p* = 0.0013, *p*-adjust = 0.05) between Starkstein Apathy Scale scores (STARK) and the ECOCAPTURE metric *non-activity* (*F_NACT*) in the freely moving (self-guided) phase for the whole population.

The core of the method consists of providing metrics (quantitative data) from raw data (video, and sensor) and then transforming them into information to propose novel marker(s) of apathy [i.e., signature(s) of behavioral syndromes]. For that, recording of behavior was made by means of a multimodal registering system including sensor and video. A multiple-camera system (*n* = 6) was set up with video coverage of the whole room (Supplementary Material-A—Figure 2). In parallel, a body sensor (Move II®, Movisens, Karlsruhe, Germany) was fixed to the subject's hip.

#### ECOCAPTURE Video-Based Behavior Metrics

The video-based behavioral metrics are generated by a manual video annotation tool, *The Observer XT*® (Noldus, Wageningen, The Netherlands), using an ethogram, or coding scheme, listing behavioral categories (such as exploration behavior and walking). *The Observer* is an observation software and a manual event recorder for the collection, management, analysis and presentation of observational data. Each record is attached to a timestamp so that the computer can produce information about the location in the timeline and the duration of any recorded behavior. *The Observer* translates the observations (videos) into computer language and exports an annotated csv file containing the frequency and duration of each categorized behavior, allowing the measurement of each behavior, and then processes them to produce statistics and graphs (Supplementary Material-C—Figures 1, 2).

To encode the videos, the rater visualized the six videos covering the different viewpoints of the waiting room as well as the ethogram resource. The ECOCAPTURE ethogram (Supplementary Material-C—Table 1) is built from two main classes: “posture” and “activity.” These classes reflect normal and abnormal behavior expected to be observed in the waiting room. Each class was composed of mutually exclusive behaviors (i.e., categories). In regard to the class called “posture,” we defined four categories: “reclining,” “sitting,” “standing,” and “walking.” In regard to the class called “activity,” there were three categories: “exploration,” “activity,” and “non-activity.” For each of these categories, the rater could select a “modifier” to specify the nature of the exploration or activity. These modifiers were items present in the environment, with which the subject could interact: books, magazines, a sofa, food, drinks, and games (Supplementary Material-C—Table 1). Additional details about how often the groups exhibited the specific activities or explorations listed in the ethogram are included in Table 2.

**Table 2:** Details about how often the groups exhibited the specific activities or exploratory behaviors listed in the ECOCAPTURE ethogram.

	**Healthy participants**	**bvFTD patients**
**Activity**		
Drinks	3	10
Games/Puzzle/Kapla	4	9
Drink preparation	5	5
Food	2	3
Books/Magazines	10	2
Radio/Speaker	3	2
Personal object	1	
Total	28	31
**Exploration**		
Groundless	23	16
Outside/Window	7	14
Games/Puzzle/Kapla	12	12
Sink		7
Books/Magazines	17	5
Furniture/Kitchen/Cooler	8	5
Sofa	8	5
Cameras	1	3
Draw unit	9	2
Personal object		2
Posters	4	2
Alarm	1	1
Chair	1	1
Door	1	1
Draw Unit/Pens	2	1
Phone	1	
Radio/Speaker	2	
Scales	1	
Table	1	
Total	99	77

Interrater reliability for encoding and categorization of behavior was tested in a significant subset of participants (*n* = 8; 4 bvFTD and 4 healthy participants). One of the two raters (a clinical research assistant) was blinded to the group identities of the subjects. The difference between raters was only 5.94% for the whole set of data (3.7% for the healthy control group and 8.1% for the bvFTD group), indicating good interrater reliability.

#### ECOCAPTURE Sensor-Based Metrics

**Figure 3:**
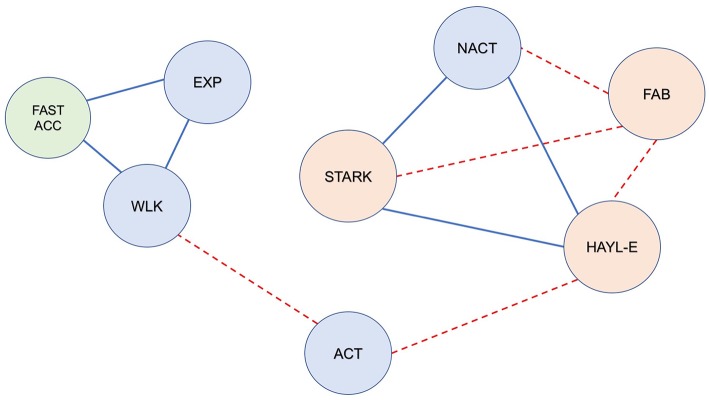
Correlations between video-based metrics, sensor-based metrics and neuropsychological data in the whole population (bvFTD + healthy participants) during the freely moving (self-guided) phase (*FULL PERIOD*). Red dotted line: significant negative correlation; solid blue line: significant positive correlation (*r, p* < 0.05). Blue circles represent the video-based metrics: EXP, *exploration*; ACT, *activity*; NACT, *non-activity*; WLK, *walking*. The green circle represents the sensor-based metric; FAST_ACC, *fast acceleration*. Orange circles represent the neuropsychological data: FAB, Frontal Assessment Battery; HAYL_E, Hayling Test; STARK, Starkstein Apathy Scale. Note the following: (1) *Exploration* and *walking* were positively correlated with each other, and both were correlated with *fast acceleration*. (2) *Activity* was negatively correlated with cognitive disinhibition (Hayling E); (3) *Non-activity* was positively correlated with apathy and cognitive disinhibition (STARK & Hayling E) and negatively correlated with FAB; (4) FAB was inversely correlated with STARK and Hayling E (which were positively correlated with each other).

**Figure 4:**
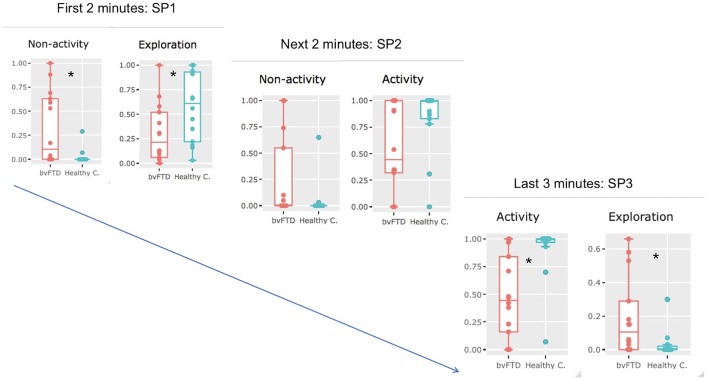
Temporal dynamics of exploration in bvFTD and healthy participants. When the full period (7′) was divided into three subperiods (*SP1, SP2*, and *SP3*), we observed differences in the successive behaviors adopted by healthy participants (healthy c.) and bvFTD patients. During *SP1* (the first 2 min), *non-activity* was significantly higher in bvFTD patients than in controls (Wilcoxon, *p* = 0.013), while *exploration* was significantly higher in healthy participants (Welch two-sample *t*-test, *p* = 0,036). During *SP2* (the next 2 min), although no significant difference was observed between the two groups for the four metrics, bvFTD patients tended to be less active (*non-activity*: *p* = 0.078), and healthy subjects tended to be more involved in activities (*activity*: *p* = 0.098). During *SP3* (the last 3 min), *activity* was significantly more important in healthy participants (Wilcoxon, *p* = 0.0015), while *exploration* was significantly higher in bvFTD (Wilcoxon, *p* = 0.012). *significant difference was observed between the two groups for the specific metrics.

**Figure 5:**
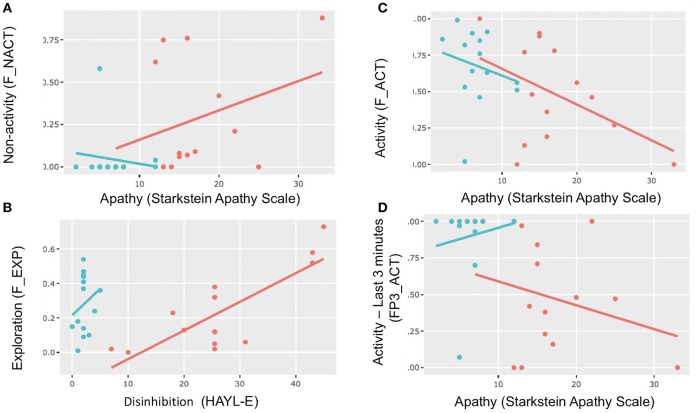
Scatterplots and correlations. Scatterplots showing correlations between apathy (Starkstein) and *activity* and *non-activity* in different periods and between disinhibition (Hayling E) and *exploration*. Red dot: bvFTD; blue dot: healthy control. **(A)** Correlations between apathy and *non-activity* in the FULL PERIOD. **(B)** Correlations between disinhibition (Hayling E) and *exploration* in the FULL PERIOD. **(C)** Correlations between apathy (Starkstein scale) and *activity* in the FULL PERIOD. **(D)** Correlations between apathy (Starkstein scale) and *activity* in SUBPERIOD 3.

The device used is a triaxial accelerometer able to record the intensity of acceleration (in multiples of *g* or units of units ms^−2^) in three mutually orthogonal directions (ACC_X, ACC_Y, ACC_Z) with a sample rate of 64 Hz with a measurement range of ±8 g (Supplementary Material-C—Figure 3). The accelerometry data belonged to another data stream and were not coded in the *Observer* system but merged later with the behavioral data. Data processing was performed with *MATLAB R2017b*. We developed *MATLAB* routines to analyze a binary file recorded by the sensor during the scenario and to calculate the metrics (3D acceleration). We processed the metrics (ACC_X, ACC_Y, ACC_Z) by the root sum squared (RSS) method and obtained the metric *acceleration* (ACC, intensity of acceleration) (Supplementary Material-C—Figure 4). The ACC data were segmented into temporal windows (according to the ECOCAPTURE scenario), and we calculated the mean of ACC during a specific time period (e.g., freely moving phase) to generate the period-dependent metric (e.g., F_ACC, mean value of ACC during the freely moving phase). Finally, we fixed several thresholds (from 0.01 to 0.9 g) to determine the time indices when the acceleration exceeds a particular threshold value and generate other metrics based on the bracketing duration (in seconds) over a specific level of acceleration (Supplementary Material-C—Figure 5). To characterize and identify the relevant threshold, we studied the fusion of video-based metrics and sensor-based metrics (Supplementary Material-C—Figure 6) (e.g., mean value of the acceleration during all the instance of walking for the whole population). We found a significant difference in the intensity of the mean acceleration between a walking state and the other physical states (standing, sitting and lying), regardless of the type of subject (healthy control or bvFTD patient) and regardless of the phase of the ECOCAPTURE scenario. Moreover, for the walking state, the third quartile value of the mean acceleration variable (ACC) was equal to 0.1 g. The average acceleration robustly differentiated a walking state from a standing or sitting state as effectively in healthy volunteers as in FTD patients. We created a novel metric, *fast acceleration*, measuring the time (in seconds) during which the acceleration signal was >0.1 g.

#### ECOCAPTURE Metrics in the Self-Guided Phase Under the Exploration Condition

Accordingly, during the first 7 min that represented the freely moving (self-guided) phase, we recorded metrics that would best describe participants' behavior in the room according to our *a priori* hypotheses that bvFTD patients would be less active than normal participants, that their exploration would be shorter, that their total duration of non-activity would be longer and that their active behavior would be reduced. This may also have consequences in terms of actimetry, with reduced activity related to movement in bvFTD patients. Consequently, we selected the four following metrics to describe behavior under the condition of exploration:

1. *Exploration*: the ratio of time spent in moving in the room or manipulating objects or remaining static but with attention oriented toward an object for more than 1 s and <10 s.
2. *Activity*: the ratio of time spent in sustained and coherent, non-automatic actions (such as manipulating objects or orienting her/his attention oriented toward an object) for more than 10 s. A time-out of 10 s is used in case of any ambiguities between the codes. In some cases, the observed behavior can be obviously considered an activity (e.g., drinking, eating) or an explorative behavior (walking, standing still near the window and looking outside). Conversely, some behaviors needed to be clarified, taking into account the duration. For example, for a stationary subject, pulling out and flipping through a book for several seconds can be considered exploration; however, beyond the time-out of 10 s, we can consider this as a reading activity.
3. *Non-activity*: the ratio of time spent stationary (standing, seated or reclining) with no apparent ongoing activity.
4. *Acceleration* (based on the 3D accelerometer data): mean acceleration during a given time window (in *g*) and *fast acceleration*, the latter of which represented the time (in seconds) during which the acceleration signal was >0.1 g.

Other metrics, such as *walking, seated* and *standing*, were also analyzed and used for correlations with the four main metrics.

### Neuropsychological Evaluation

#### Apathy Scales

“Classic” apathy assessment was performed with the Starkstein Apathy Scale, filled out by caregivers and patients (24), and the Dimensional Apathy Scale (DAS) (25), which consists of three subscales (initiation, cognition and emotion) based on the theoretical model of three forms of apathy according to Levy and Dubois (1). These scales were filled out by the participants in the presence of the examiner (a neuropsychologist) to ensure that the questionnaires were correctly understood and filled out.

#### Other Neuropsychological Tests

The Mini-Mental State Evaluation (MMSE) was used to determine general cognitive efficiency (26). Frontal and executive functions were globally assessed with the Frontal Assessment Battery (FAB) (27, 28). The Hayling Sentence Completion Test (HSCT, the “Hayling test”) has been used to evaluate cognitive disinhibition (29). This test measures response initiation and inhibition of response by a sentence completion task. It is made of two subtasks: in section A (“Hayling A”), subjects have to complete a sentence in an automatic condition, with an appropriate word (for instance, for ≪ The rich child attended a private … ≫, correct answer is ≪ school≫). In section B (“Hayling B”), subjects have to complete a sentence in an inhibition condition, with an inappropriate word, needing inhibition of automatic answer (for the sentence ≪ London is a very lively… ≫, ≪ city ≫ is an incorrect answer, but ≪ banana ≫ is a correct answer). Cognitive disinhibition is usually assessed by subtracting the *total latency time* in section B from the *total latency time* of A (“Hayling B-A”). Errors in section B are recorded and form an error score (“Hayling E”). The Mini-SEA (Social Cognition and Emotional Assessment) evaluates deficits in emotion recognition and theory of mind, both recognized as hallmark features of bvFTD (30, 31). Depression and anxiety were explored with the Hospital Anxiety and Depression (HAD) Scale (32).

### Statistical Analyses

The free-moving phase was analyzed as a single period of time with a total duration of 7 min (*FULL PERIOD*). We hypothesize that exploration behavior may evolve during the 7-min period. Could the very first minutes in the room differ from the last minutes of the time dedicated to exploring the room? To detect potential changes/switches in the dynamic of behavior during this freely moving phase, we divided it into three successive periods: the first 2 min (*SUBPERIOD* 1), when the participants entered the room and discovered it; the next 2 min (*SUBPERIOD 2*); and the last 3 min (*SUBPERIOD 3*).

BvFTD patients and healthy participants were compared in terms of four main metrics (*exploration, activity, non-activity* and *acceleration*) for the *FULL PERIOD* and for each of the three *SUBPERIODs*.

First, between-group comparisons were made for each of the four metrics as well as for apathy scales and other neuropsychological data using *R* software (version 3.4.3, R Studio, Inc.). To run parametric tests on the data, we first checked the normality assumption with a Shapiro test and then tested the homogeneity of group variance (homoscedasticity condition) with Levene's test. When the condition of population normality was verified, we used Welch's two sample *t*-test (“Welch's *t*-test for unequal variances”) to compare the means of the two samples. Welch's *t*-test is designed for unequal variances and unequal sample sizes that maintain the assumption of normality. Therefore, whether the homoscedasticity condition was verified or not, we used Welch's *t*-test to compare the means of two samples. When the data samples did not follow a normal distribution, we used the Mann-Whitney-Wilcoxon test to compare the ranks.

Second, correlation analyses were performed for all the data obtained.

The pathological context may disrupt the continuity of data, and the differences in performance between groups may mask the variance of performance within a given group. Correlations were performed for the whole population (bvFTD patients and healthy participants in the same matrix) and for bvFTD patients. The Holm–Bonferroni method was used to adjust the *p*-value in the case of multiple comparisons.

We used the Pearson correlation coefficient to measure linear correlation between two variables in parametric tests when the normality of distribution by subgroup was verified. When the data samples did not follow a normal distribution, we use the Spearman coefficient (r) to build the correlation matrix. When the *p*-value was below the significance level (*p* < 0.05), we rejected the null hypothesis, which posited no linear relationship between the 2 variables.

In order not to delete either individuals or variables from the analysis because of a few missing data, we estimated the missing values as the median of each of the variables (“imputation by the median”).

## Results

### Neuropsychological Testing

#### Apathy in bvFTD

A significant difference was observed for the Starkstein Apathy Scale (bvFTD: 17± 6.39; HC: 6.71 ± 2.76; Welch *t*-test, *p* = 0.00003) between bvFTD patients and HC. Ten out of 14 bvFTD patients were above the Starkstein pathological cut-off (14/42), while no HC were above this threshold (Figure 2). No significant difference was found for the DAS total score. Only the DAS-cognition subscore was significantly different between the two groups (Welch *t*-test, *p* = 0.0016) but not for the two other subscores (initiation and emotion) (Table 1).

#### Disinhibition in bvFTD

Hayling E was significantly higher in bvFTD than in HC (Wilcoxon, *p* = 5.21e-06) (see Table 1).

#### Other Tests and Scales

BvFTD patients presented with a significant decrease in global cognitive efficiency (MMSE: bvFTD: 22.93 ± 2.4 [range: 20–29], HC: 29.64 ± 0.63 [range: 28–30]; Wilcoxon, *p* = 7.49e-06) and a sharp frontal syndrome as revealed by the FAB score (bvFTD: 12 ± 3.55, HC: 17.57 ± 0.65; Wilcoxon, *p* = 4.03e-05) (Table 1). SEA (theory of mind and facial emotion recognition), previously shown to discriminate bvFTD from other conditions such as AD or depression (30, 31), was impaired in bvFTD patients (bvFTD: 18.38 ± 3.17; HC: 26.63 ± 1.11, Welch *t*-test, *p* = 8.30e-08). In both groups, no anxiety was detected by the HAD-A, while a higher level of depression was present in bvFTD patients than in HC as shown by the HAD-D (bvFTD: 6.14 ± 3.37, healthy participants: 1.36 ± 1.9); Wilcoxon, *p* = 0.0003).

### Video and 3D Acceleration Analyses During the Freely Moving (Self-Guided) Phase

#### The Freely Moving Phase as a Whole (FULL PERIOD)

Here, we compared bvFTD and HC using the four predefined metrics (*exploration, activity, non-activity* and *acceleration*) during the full 7-min period when the participants entered the room and were encouraged to explore it. We found that in bvFTD patients, a significant part of the time spent in the room was occupied by *non-activity*, which was not the case for HC (ratio of time represented by *non-activity*: bvFTD: 0.28 ± 0.33 [range: 0.00–0.88], HC: 0.04 ± 0.15 [range: 0.00–0.58]; Wilcoxon, *p* = 0.002) (Figure 2). Most of the bvFTD patients, entering in the room, went straight to a chair or to the sofa and then remained still for more than a minute. None of the HCs exhibited such behavior. In addition, there is a significant positive correlation (*r* = 0.58, *p* = 0.0013, *p*-adjust = 0.05) between Starkstein Apathy Scale scores and the ECOCAPTURE metric *non-activity* for the whole population.

The three additional metrics (*exploration, activity* and *acceleration*) were not found to be significantly different between the two groups.

In addition, in the whole matrix (bvFTD and HC), *exploration* was positively correlated with *fast acceleration* (*r* = 0.57, *p* = 0.0016, *p*-adjust = 0.05) and *walking* (*r* = 0.70, *p* = 3.2e-05, *p*-adjust = 0.0016) (Figure 3). In the bvFTD matrix, *exploration* was positively correlated with *fast acceleration* (*r* = 0.59, *p* = 0.026, *p*-adjust = 0.05).

We performed further analyses to verify whether a particular time dynamic of behavior, masked by the global analysis, could be extracted. For that, we segmented the *FULL PERIOD* phase into three subperiods (the first 2 min [*SUBPERIOD 1*], the next 2 min [*SUBPERIOD 2*] and the last 3 min [*SUBPERIOD 3*]) (Figure 4).

#### Subdivision of the Freely Moving Phase Into Three Periods (Figure 4)

During *SUBPERIOD 1*, we observed that two metrics discriminated the two groups of participants most effectively: *non-activity* was significantly more common in bvFTD patients than in HC (Wilcoxon, *p* = 0.013), while *exploration* was significantly higher in HC than in bvFTD patients (Welch two-sample *t*-test, *p* = 0.036).

During *SUBPERIOD 2*, no significant difference was observed between the two groups for the four metrics, although the bvFTD patients tended to be less active than HC (*non-activity*: *p* = 0.078) and the HC tended to be more involved in activities (*activity*: *p* = 0.098).

During *SUBPERIOD 3*, two metrics discriminated between the two groups of participants most effectively: *activity* was significantly more common in HC than in bvFTD patients (Wilcoxon, *p* = 0.0015), while the opposite was true of *exploration* (Wilcoxon, *p* = 0,012).

In summary, bvFTD patients were mostly characterized by *non-activity* in *SUBPERIOD 1* and *exploration* in *SUBPERIOD 3*, while healthy participants were mostly characterized by *exploration* in *SUBPERIOD 1* and *activity* in *SUBPERIOD 3* (Figure 4).

### Correlations Between Video and Sensor-Based and Neuropsychological Data

For *FULL PERIOD*, we found that in the whole population matrix, *non-activity* was correlated with apathy (Starkstein Apathy Scale, *r* = 0.58, *p* = 0.0013, *p*-adjust = 0.05) (Figure 5). In addition, apathy (Starkstein Apathy Scale) was negatively correlated with frontal syndrome (FAB, *r* = −0.73, *p* = 1.03e-05, *p*-adjust = 0.00054) and positively correlated with cognitive disinhibition (Hayling E, *r* = 0.79, *p* = 5e-07, *p*-adjust = 2.77e-05) (Figure 3).

In the bvFTD matrix, we found that *exploration* was correlated with cognitive disinhibition in the *FULL PERIOD* (Hayling E, *r* = 0.66, *p* = 0.0106, *p*-adjust = 0.042) as well as in S*UBPERIOD* 2 (Hayling E, *r* = 0.73, *p* = *0.003, p*-adjust = 0.017). In addition, in the whole population matrix, during SUBPERIOD 3, *activity* was inversely correlated with Hayling E (*r* = −0.59, *p* = 0.0403, *p*-adjust = 0.041).

## Discussion

In this study, we observed and analyzed the behavior of a group of bvFTD patients and a group of healthy participants in an ecological setting, with the main aim of objectively measuring apathy.

As expected, bvFTD patients were apathetic according to their score on the classical Starkstein Apathy Scale. When exposed to a new environment (ecological experimental setting), instead of interacting with it, bvFTD patients remained inactive, standing still, seating on a chair or lying on the sofa, contrasting with healthy participants who explored and interacted with this new environment. As *non-activity* strongly correlates with apathy scores (on the Starkstein Apathy Scale), inactivity can be considered as part of an apathetic syndrome mostly characterized by a deficit of self-initiated voluntary actions and a quantitative decrease in sustained goal-directed actions. In addition, in bvFTD patients *exploration* was decreased compared to healthy participants and it was correlated with cognitive disinhibition and not with 3D acceleration, in contrast to healthy participants. Thus, exploration in bvFTD patients moved away from normality and seemed to reflect cognitive disinhibition. Together, these findings demonstrated a deficit of exploration behavior in bvFTD patients.

In the exploratory behavior of bvFTD patients, we observed a different temporal pattern from that of healthy participants. Indeed, upon dividing the whole freely moving phase into three successive phases, we observed that the time dynamic of behavior for healthy participants started with an exploration phase. Then, healthy participants were progressively involved in more sustained activities. In contrast, bvFTD patients were inactive at first; only at the end of the freely moving phase did they start to explore the room.

The correlations in the whole population matrix between *non-activity*/*exploration* and more classic neuropsychological tests or scales evaluating apathy, disinhibition and dysexecutive functions showed that exploration deficit was part of a frontal syndrome.

To our knowledge, this study was among the first attempts to objectively characterize apathy in a semi-ecological context. Previously, Müller and colleagues used actigraphy in real-life situations and showed a reduced number of activities in everyday life in apathetic patients (33). In the present study, because of its novelty, the methodological approach may require improvements, and some limitations should be taken into account. Videos and sensors generated many potentially analyzable data, and other metrics could have been as relevant as the ones selected. However, we also analyzed more direct and obvious metrics, such as “*walking*,” “*reclining*,” and “*stand still*” (the ratio of time spent in each of these physical postures). We showed that *exploration* was strongly and positively correlated with “*walking*” (as well as with *fast acceleration*). These correlations reinforced the relevance of the way we have defined and chosen the different metrics of interest. Indeed, it seems logical that *exploration* was strongly correlated with *walking* and *acceleration* (it is expected that subjects will move and walk in order to explore the room), while being involved in sustained activities required patients to stop walking and not recline on the sofa or remain standing on their feet.

Moreover, we should acknowledge that the sample size was relatively small. Nevertheless, the subjects' behavior was significantly different between the two groups.

Furthermore, the authors were not blinded to the diagnosis when categorizing behavior (activity, non-activity, exploration and acceleration). This may be considered a bias in the analysis. However, it is important to note that, because of (i) of the precise definition of each metric and, for some of them, an additional associated variable such as a duration; (ii) the automatic reporting of each metric on an ethogram-based graph; (iii) the high level of category match between raters in a significant sample of the studied populations; and (iv) the sharp group differences in these very metrics, it is very unlikely that this bias had more than a marginal effect on the results.

Although it was clear that bvFTD exhibited exploration deficits, the specific underlying mechanisms behind exploration and its deficits are difficult to identify. Nevertheless, future analyses, such as the study of the other phases of this multiple-step scenario, may disambiguate these mechanisms. Consequently, at this stage of the advancement of this research protocol, we acknowledge that the following discussion contains speculations regarding the plausible mechanisms underlying exploration deficits.

How can we explain the fact that bvFTD patients were mostly inactive while normal subjects explored the new location and then engaged in sustained activities? First and obviously, *non-activity* can be considered a direct clinical expression of apathy [i.e., a quantitative decrease in goal-directed actions according the consensus definition of apathy (2)]. In support of this idea, *non-activity* was strongly correlated with the Starkstein Apathy Scale. In the present experimental scenario, participants were not asked to perform specific actions; rather, they were left with a certain degree of freedom/choice in which voluntary, exploratory behavior should be self-generated. Therefore, we may consider this situation favorable for generating voluntary, willed, self-guided behavior. Consequently, *non-activity* probably reflected the inability of bvFTD patients to self-generate goal-directed behavior, as expected given their apathy.

Four psychological/cognitive mechanisms could be discussed to explain the decrease in self-initiated exploration observed in bvFTD: (1) The lack of interest reflects a dysfunction of the reward valuation system (a “motivation” deficit) (18). Here, to reinforce exploration and interactions with the in-room items, we customized the room with each participant's favorite food, drinks, magazine reading and music. Accordingly, one should expect that in the context of bvFTD and frontal lesions, this would encourage an approach or utilization behavior (34, 35). This has not been observed in our group of bvFTD patients. Such a default of the valuation system has been clearly demonstrated in bvFTD patients as well as in human and non-human primates with ventromedial focal lesions (19, 36–38). (2) Regardless of their reward valuation abilities, bvFTD patients would have an inability to transfer this value into action. This would represent what has been conceptualized as a very intense form of decreased initiation, called autoactivation deficit (39, 40). Autoactivation deficit (AAD) consists of a loss of spontaneous apparent activity. Like bvFTD patients in the room, AAD patients tend to remain quietly in the same place or position all day long, without speaking or taking any spontaneous initiative. External stimulation may partially and temporarily reverse AAD, emphasizing the sharp contrast between the drastic quantitative reduction in self-generated actions and the relatively normally executed externally driven behaviors. AAD is in general due to bilateral focal lesions in the basal ganglia affecting its “limbic” and “cognitive” territories (40–42). In a previous study, we showed that in AAD due to basal ganglia lesions, the limbic and executive networks were disconnected because of the lesions; thus, the affective value of a given context does not influence decision-making (42). AAD has also been reported after frontal lesions, and it bears similarities with the clinical syndromes observed after damage to the mesial prefrontal cortex in both human and non-human primates, such as “akinetic mutism,” “motor transcortical aphasia,” or “motor neglect” (43–49). In humans, functional neuroimaging studies further support the role of the mesial prefrontal cortex in self-initiation (50–54). In the monkey, lesions of the mesial prefrontal cortex are associated with a sharp decrease in self-initiation of voluntary movements, contrasting with the total sparing of externally triggered actions (55, 56). In bvFTD, the neurodegenerative process constantly affects the mesial prefrontal cortex (20, 57, 58) and often the basal ganglia, in particular the territories where lesions induced AAD, such as the head of caudate nuclei (59, 60). Furthermore, in bvFTD, initiation deficit and apathy have been related to atrophy in the medial prefrontal cortex and in particular in the anterior cingulate cortex (61, 62). Altogether, we infer that the *non-activity* observed in bvFTD patients is quite similar to that observed in AAD, and we propose that the exploration deficit in bvFTD could be, at least partially, the clinical expression of AAD. (3) The observation that healthy subjects were involved in sustained activities, but not bvFTD patients, raised a third hypothesis to explain exploration deficits in bvFTD patients. Sustained activities required to maintain and manipulate goals and subgoals in a mental representational space (i.e., in working memory). Working memory impairment is associated with frontal lesions (63) and is part of the executive syndrome seen in bvFTD and related to apathy in this disease (19, 64). However, although possible, the working memory deficit seems to us less plausible than the two other above hypotheses. Indeed, in healthy participants, sustained activities occurred after a phase of exploration. The items in sight in the room triggered exploration behavior, and therefore exploration did not require any planning or long-term maintenance of goals and subgoals. (4) Finally, a “social cognition” hypothesis could be discussed. Indeed, one may argue that social norms would restrain one from exploring in a situation that mimics waiting in a waiting room for staff, especially while being filmed. However, normal participants (supposed to have normal social cognition) explored the room. One would expect bvFTD patients to transgress the social conventions that would lead them to behave in an appropriate manner in a social context or, conversely, to be too conformist and follow the instructions to the letter (i.e., to explore the room) (65–68). Neither situation occurred in bvFTD.

At the end of the freely moving phase, exploration behavior did eventually occur in bvFTD patients. However, it differed from that of healthy participants in several ways: it was delayed and followed a long period of *non-activity*, it was no longer correlated with 3D acceleration (significant correlation in *FULL PERIOD* but not in *SUBPERIOD 3*), and it was correlated with cognitive disinhibition. The correlation between *exploration* and *acceleration* could be interpreted as frank movements toward a given object, reflecting the clear-cut will to direct one's attention/movements toward that object. The absence of acceleration and the correlation with cognitive disinhibition could suggest that in bvFTD, patients' way of exploring the environment is more irregular and dispersive, less efficient, shorter lived, less motivated and more automatic.

It is noteworthy that in bvFTD, disinhibition and apathy may arise from dysfunctions of common fundamental processing and share the same neural bases (69, 70). This may suggest that in our experiment, in bvFTD, *non-activity* and *exploration* were the “two sides of the same coin.” Indeed, these two metrics were strongly correlated with markers of frontal/dysexecutive syndrome.

In summary, we have shown for the first time that bvFTD patients present an exploration deficit. This pattern of behavior may define apathy in bvFTD patients facing a new environment. Exploration deficits were mostly characterized by inactivity and delayed exploration. This particular pattern of behavior may reflect the interactions of three underlying pathological mechanisms: lack of motivation, autoactivation deficits and disinhibition. The analyses of the other phases of the scenario and neuroimaging data will help us to disambiguate the main mechanisms at play in apathy in bvFTD and their neural bases. Regarding medical practice, this novel way to assess apathy with objective markers can be easily transferred into operational clinical evaluation in two ways: “at the bedside” (for instance, how a patient explores a new environment such as her/his hospital room when first checking in) and “at home” with specific telemetry programs, coupling multimodal recording approaches with automatic recognition and categorization of behaviors. These two new approaches are currently under investigation. Ultimately, we would like to emphasize the importance of developing new tools for the simple, objective assessment of behavioral changes in neurological diseases, particularly FTD. Indeed, these first results pave the way for simple and objective assessment of behavioral changes, which represents a critical step toward evaluating disease progression and treatment efficacy in bvFTD.

## Ethics Statement

This study is part of clinical trial C16-87 sponsored by INSERM. It was granted approval by the national Ethics Committee, or Comité de Protection des Personnes, on 17/05/2017 and registered in a public clinical trial registry (clinicaltrials.gov: NCT03272230, NCT02496312). All study participants gave their written informed consent to participate, in line with French ethical guidelines.

## Author Contributions

RL: study concept and assessment of apathy under ecological conditions. BB, KL, JF-V, CA, BD, and RL: experimental design and ECOCAPTURE scenario. BB: data acquisition system design, sensor-based data analysis, and data management. BB, KL, and RL: ECOCAPTURE ethogram. BB and AR-L: video annotation. KL, JF-V, BB, DB, RM, AR-L, and RL: study experimental investigation and data collection. BB, VG, KL, JF-V, and RL: statistics and data analysis. DB, RM, and RL: clinical evaluations. DB, RM, RL, and AR-L: patient recruitment. AR-L: clinical trial monitoring.

### Conflict of Interest Statement

The authors declare that the research was conducted in the absence of any commercial or financial relationships that could be construed as a potential conflict of interest.
