# Supplementary material for: Exploration Deficits Under Ecological Conditions as a Marker of Apathy in Frontotemporal Dementia
Source: Front Neurol. 2019 Aug 28;10:941. doi: 10.3389/fneur.2019.00941 (PMC6736613; doi:10.3389/fneur.2019.00941)
Supplement: Supplementary file 1 [file Presentation_1.PPTX]

## Slide 1
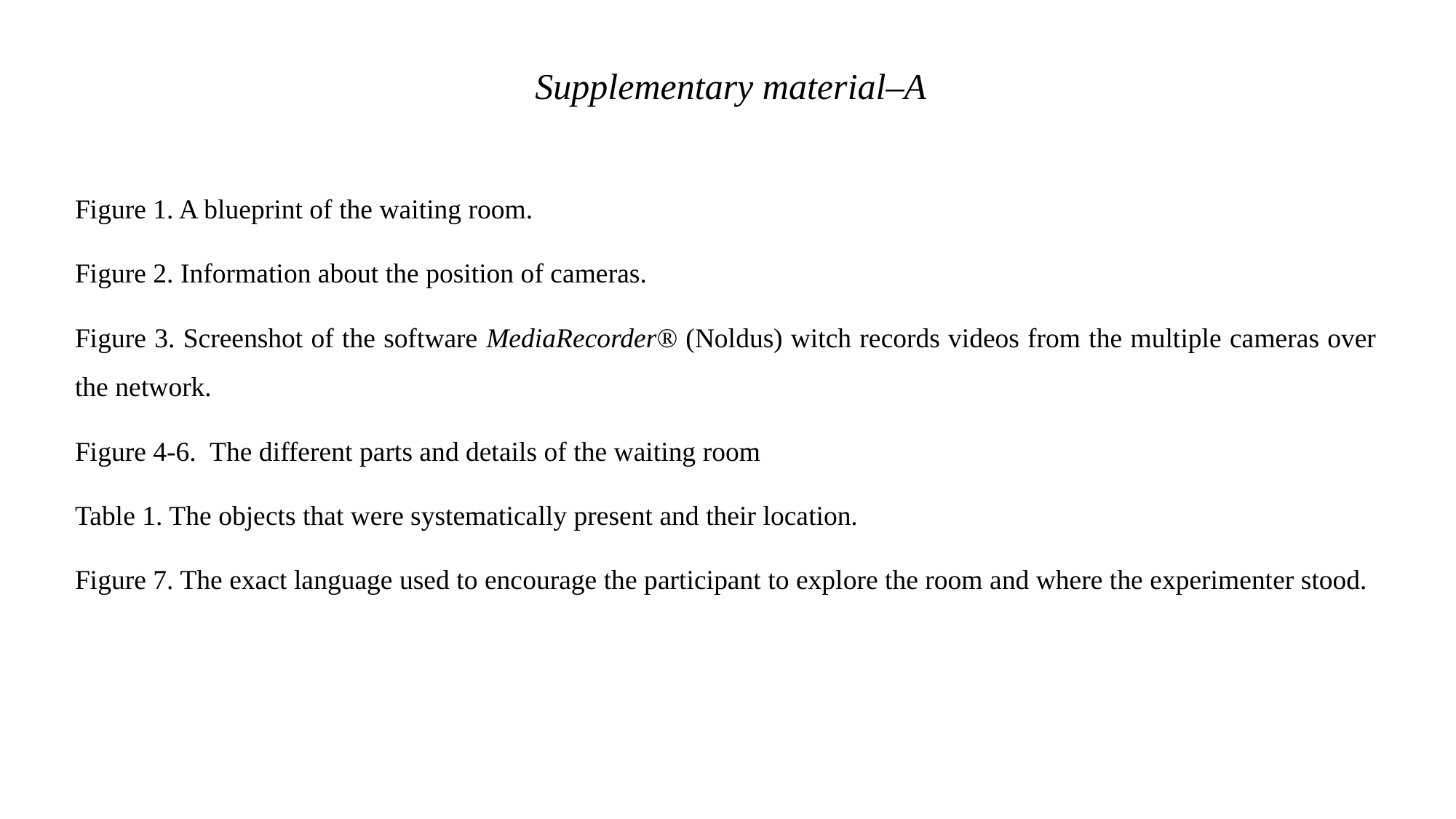

# Supplementary material–A
Figure 1. A blueprint of the waiting room.
Figure 2. Information about the position of cameras.
Figure 3. Screenshot of the software MediaRecorder® (Noldus) witch records videos from the multiple cameras over the network.
Figure 4-6. The different parts and details of the waiting room
Table 1. The objects that were systematically present and their location.
Figure 7. The exact language used to encourage the participant to explore the room and where the experimenter stood.

## Slide 2
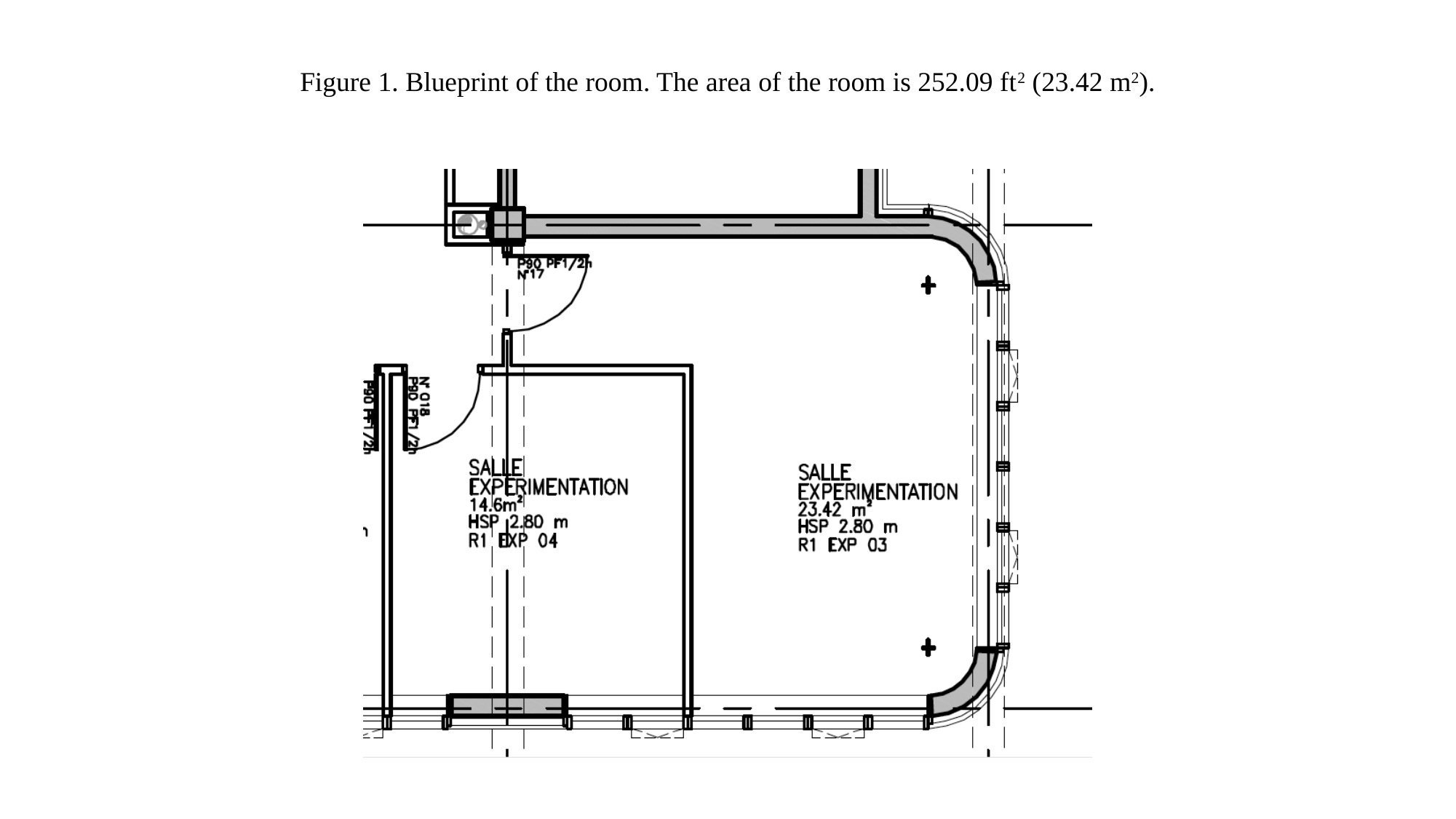

# Figure 1. Blueprint of the room. The area of the room is 252.09 ft2 (23.42 m2).

## Slide 3
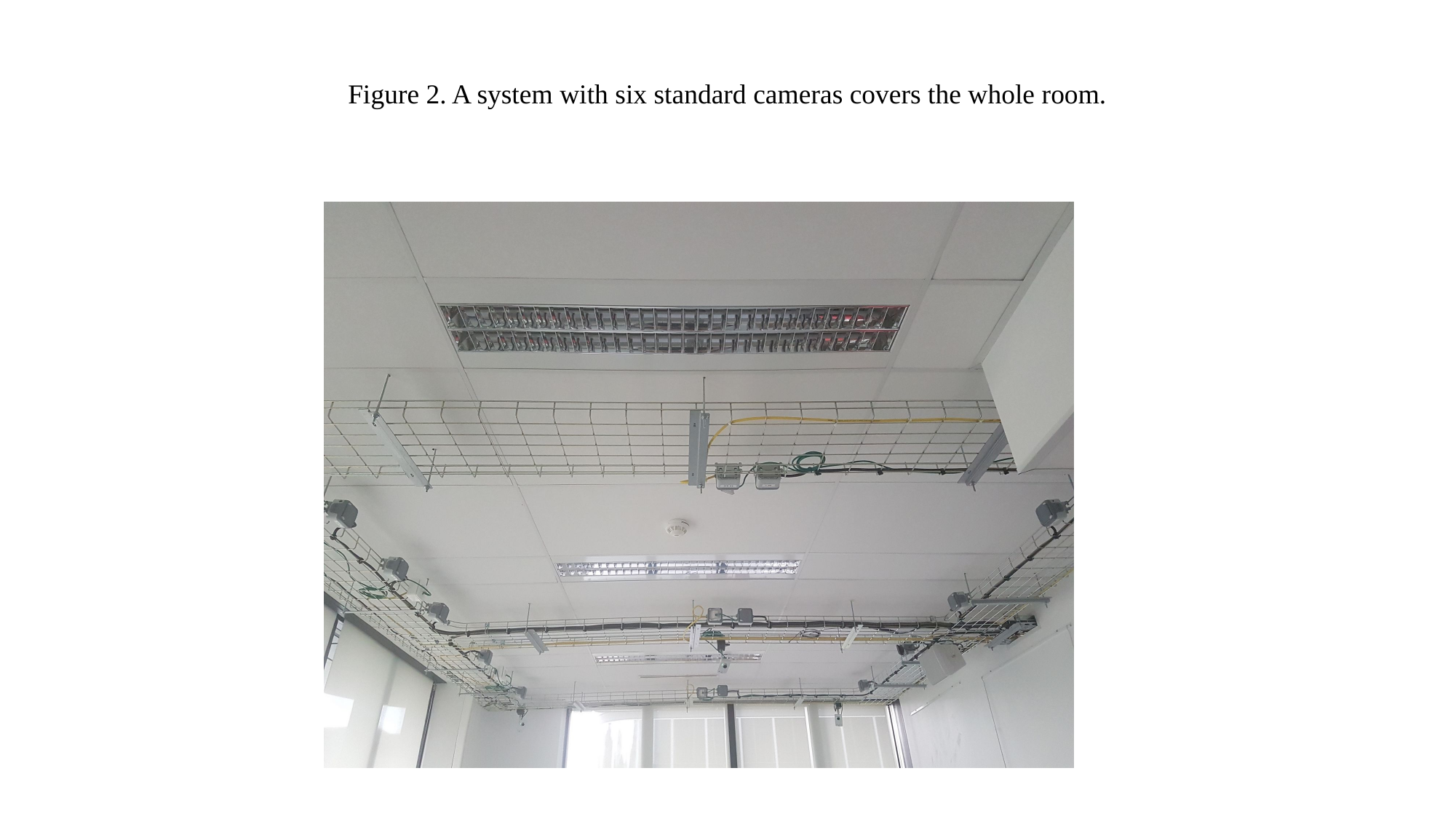

# Figure 2. A system with six standard cameras covers the whole room.

## Slide 4
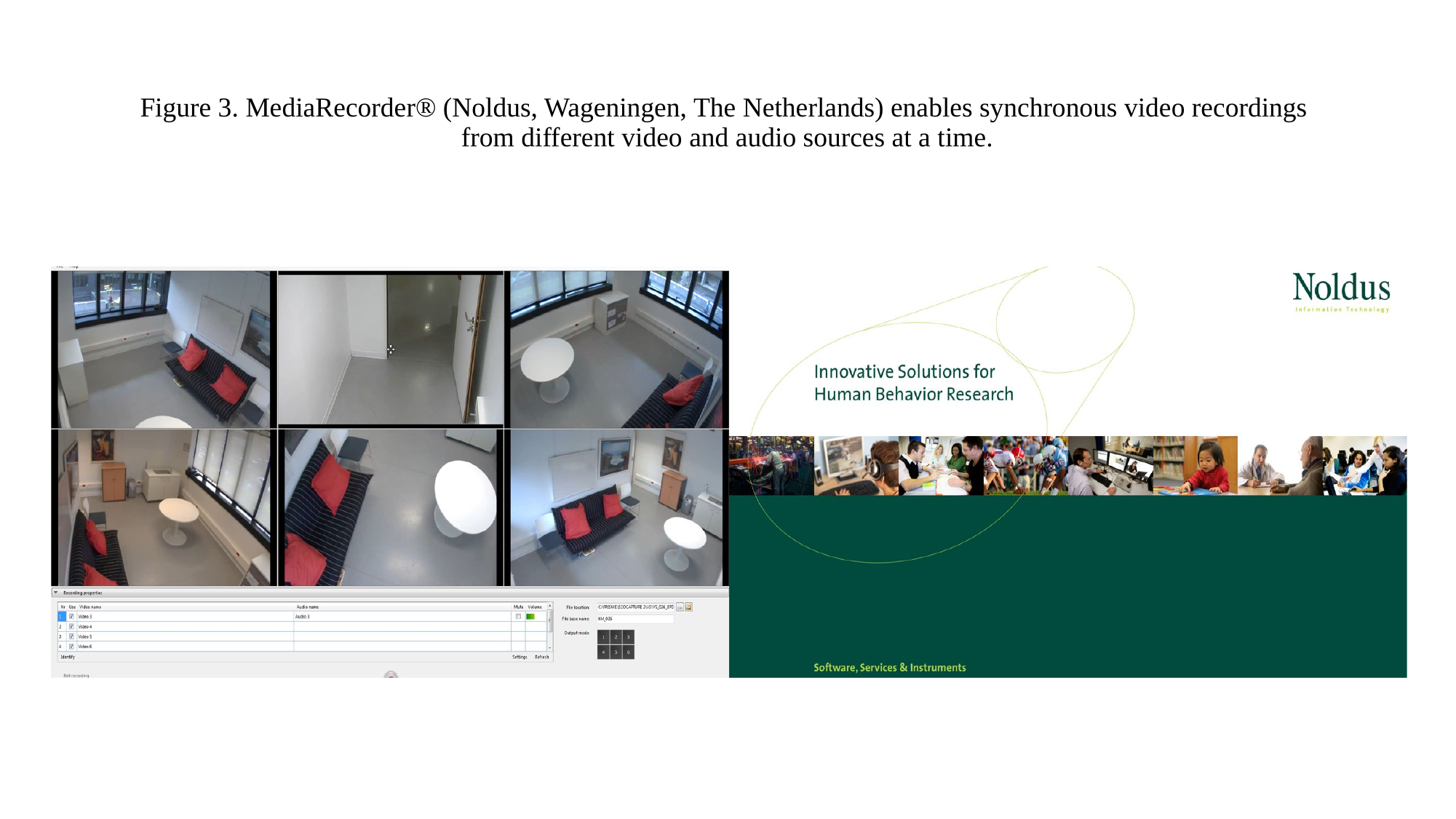

# Figure 3. MediaRecorder® (Noldus, Wageningen, The Netherlands) enables synchronous video recordings from different video and audio sources at a time.

## Slide 5
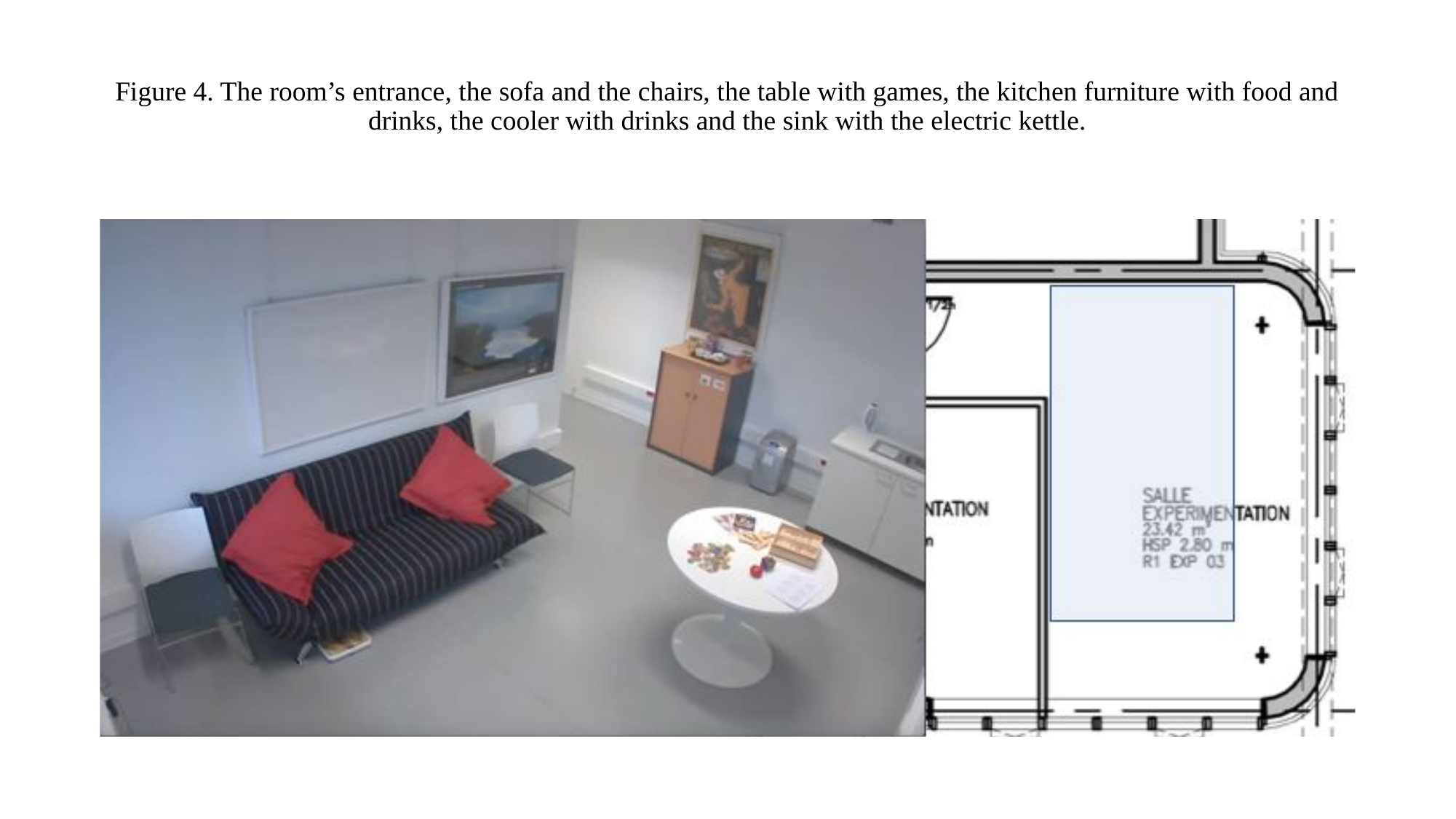

# Figure 4. The room’s entrance, the sofa and the chairs, the table with games, the kitchen furniture with food and drinks, the cooler with drinks and the sink with the electric kettle.

## Slide 6
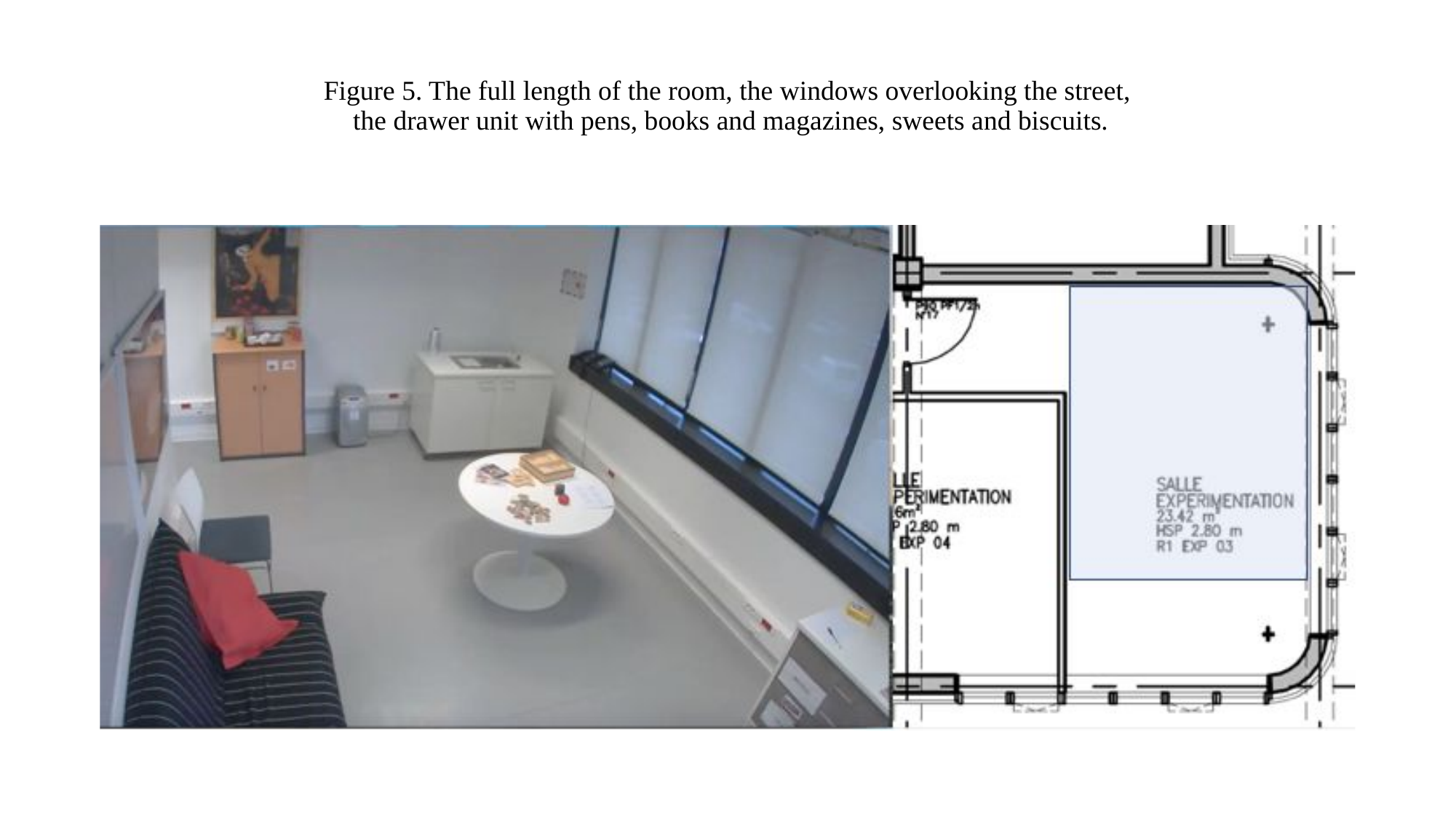

# Figure 5. The full length of the room, the windows overlooking the street, the drawer unit with pens, books and magazines, sweets and biscuits.

## Slide 7
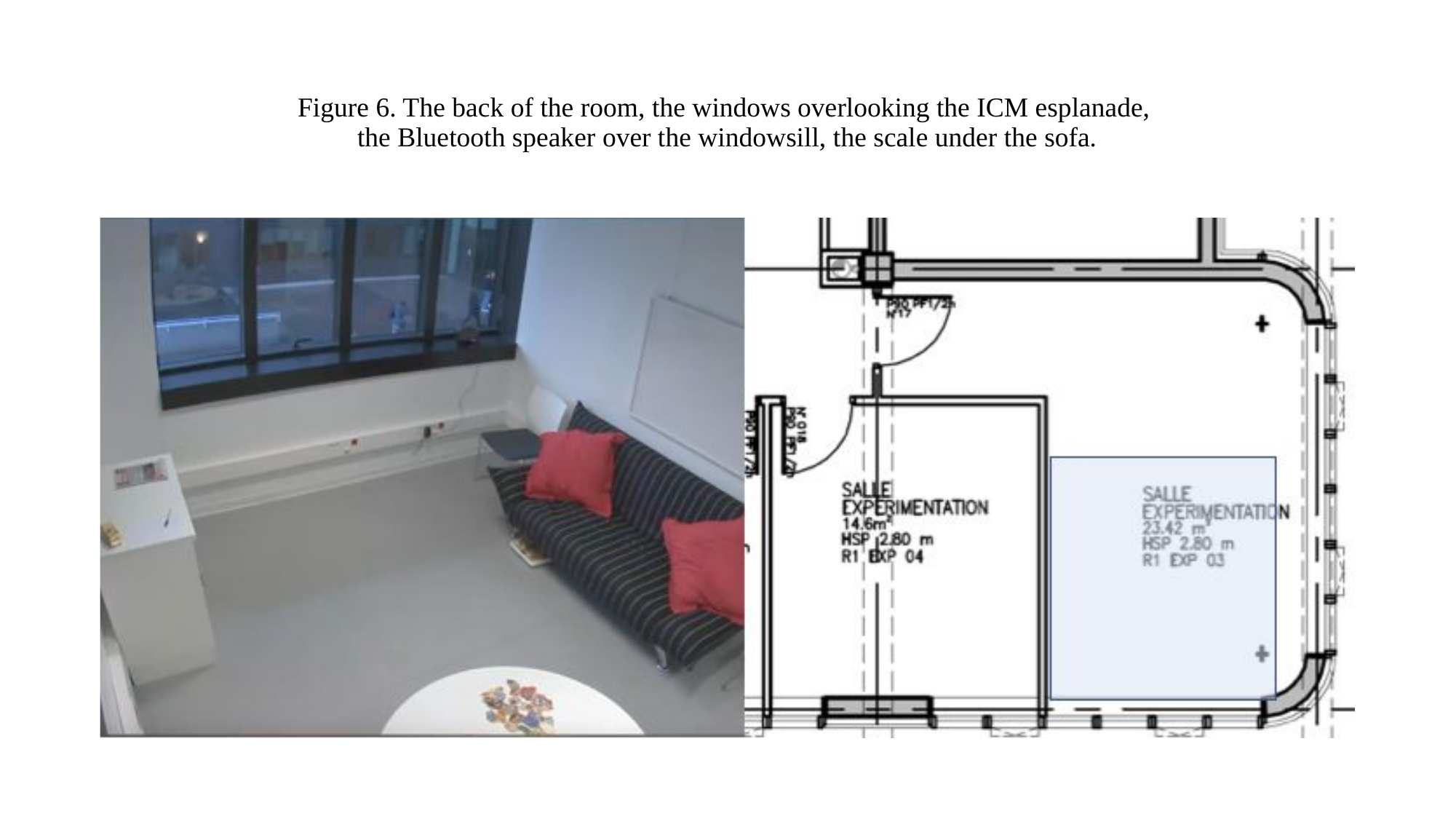

# Figure 6. The back of the room, the windows overlooking the ICM esplanade, the Bluetooth speaker over the windowsill, the scale under the sofa.

## Slide 8
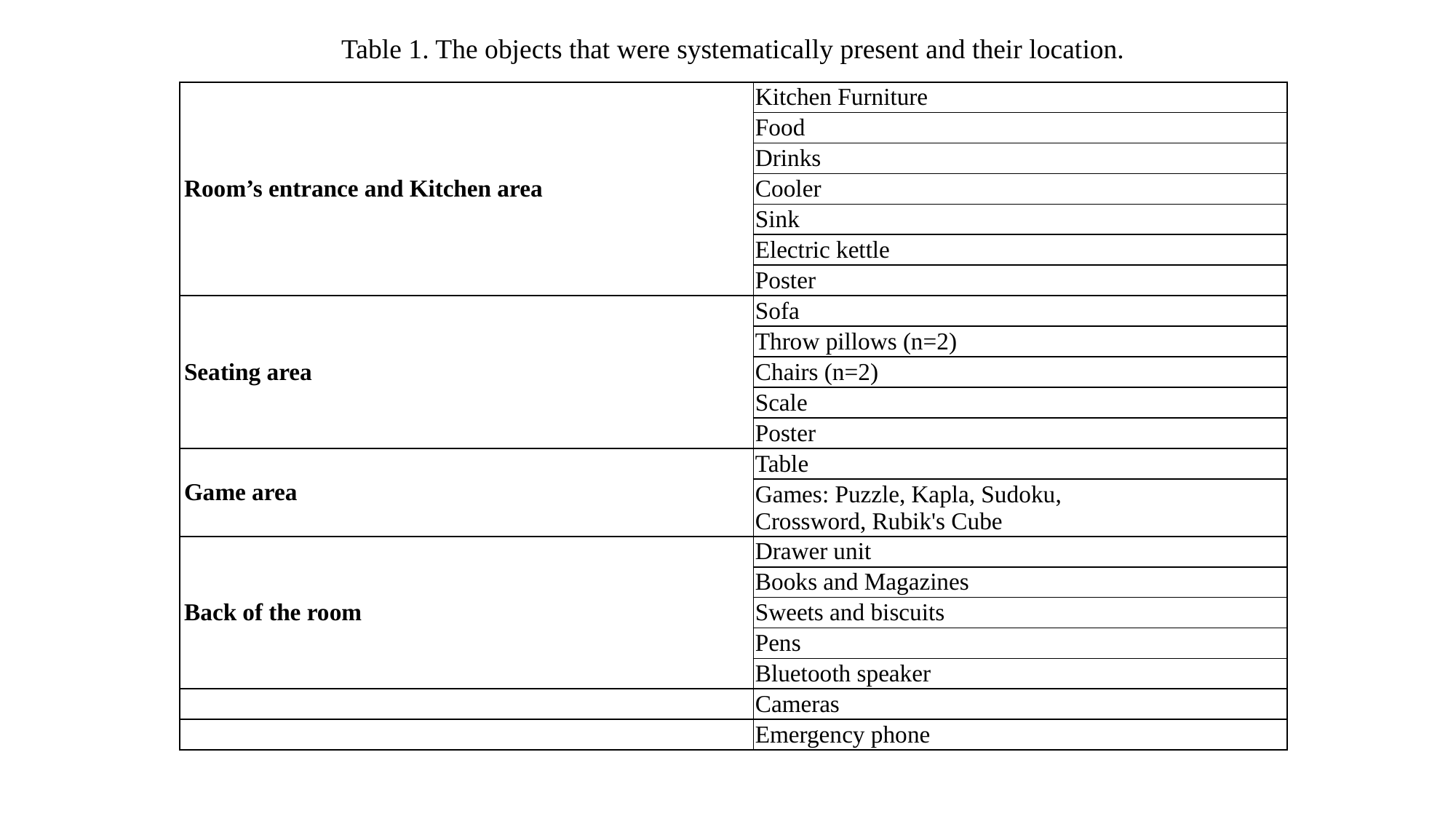

# Table 1. The objects that were systematically present and their location.
| Room’s entrance and Kitchen area | Kitchen Furniture |
| --- | --- |
| | Food |
| | Drinks |
| | Cooler |
| | Sink |
| | Electric kettle |
| | Poster |
| Seating area | Sofa |
| | Throw pillows (n=2) |
| | Chairs (n=2) |
| | Scale |
| | Poster |
| Game area | Table |
| | Games: Puzzle, Kapla, Sudoku, Crossword, Rubik's Cube |
| Back of the room | Drawer unit |
| | Books and Magazines |
| | Sweets and biscuits |
| | Pens |
| | Bluetooth speaker |
| | Cameras |
| | Emergency phone |

## Slide 9
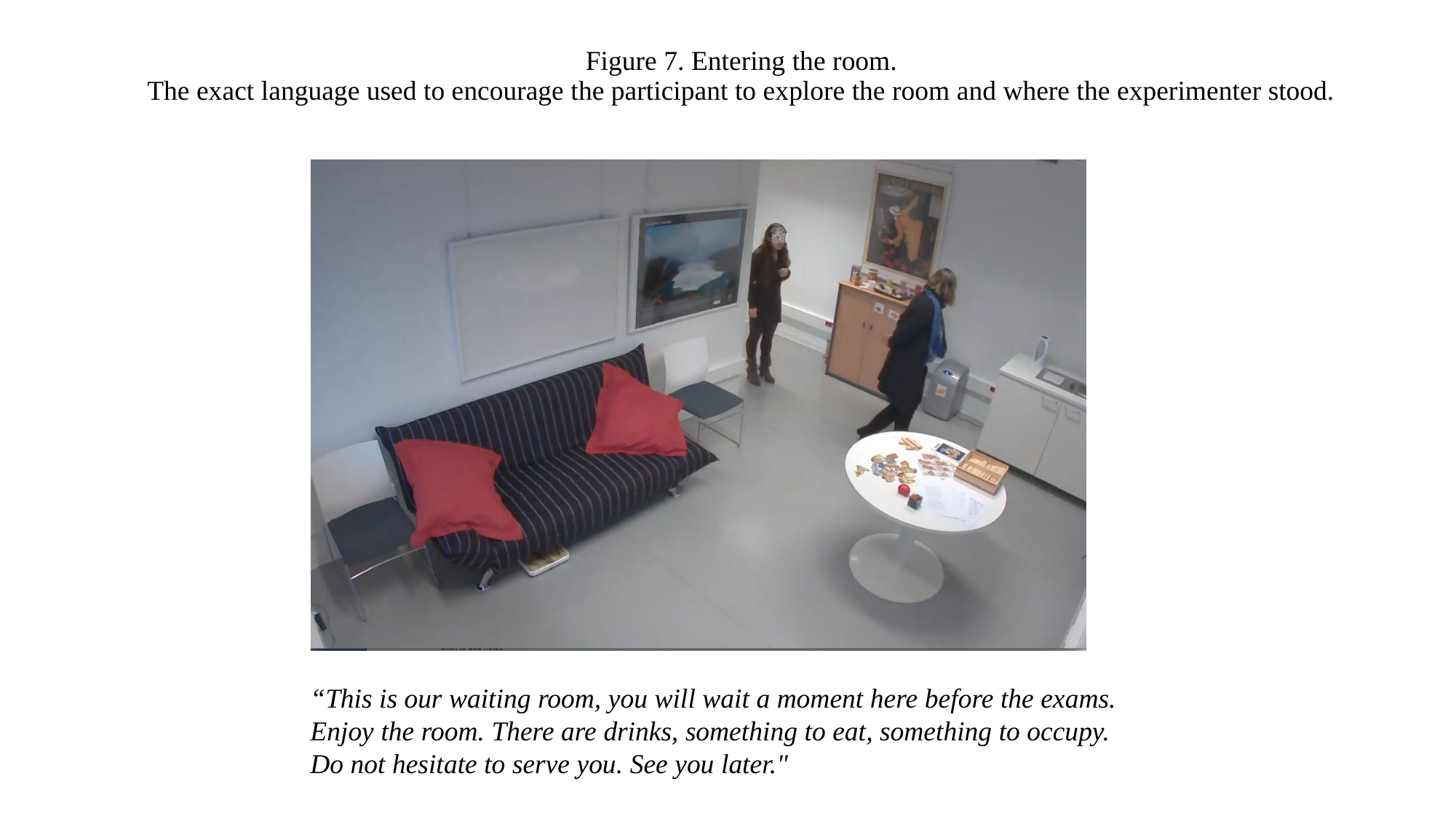

# Figure 7. Entering the room. The exact language used to encourage the participant to explore the room and where the experimenter stood.
“This is our waiting room, you will wait a moment here before the exams.
Enjoy the room. There are drinks, something to eat, something to occupy.
Do not hesitate to serve you. See you later."
