# Supplementary material for: Exploration Deficits Under Ecological Conditions as a Marker of Apathy in Frontotemporal Dementia
Source: Front Neurol. 2019 Aug 28;10:941. doi: 10.3389/fneur.2019.00941 (PMC6736613; doi:10.3389/fneur.2019.00941)
Supplement: Supplementary file 2 [file Presentation_2.PPTX]

## Slide 1
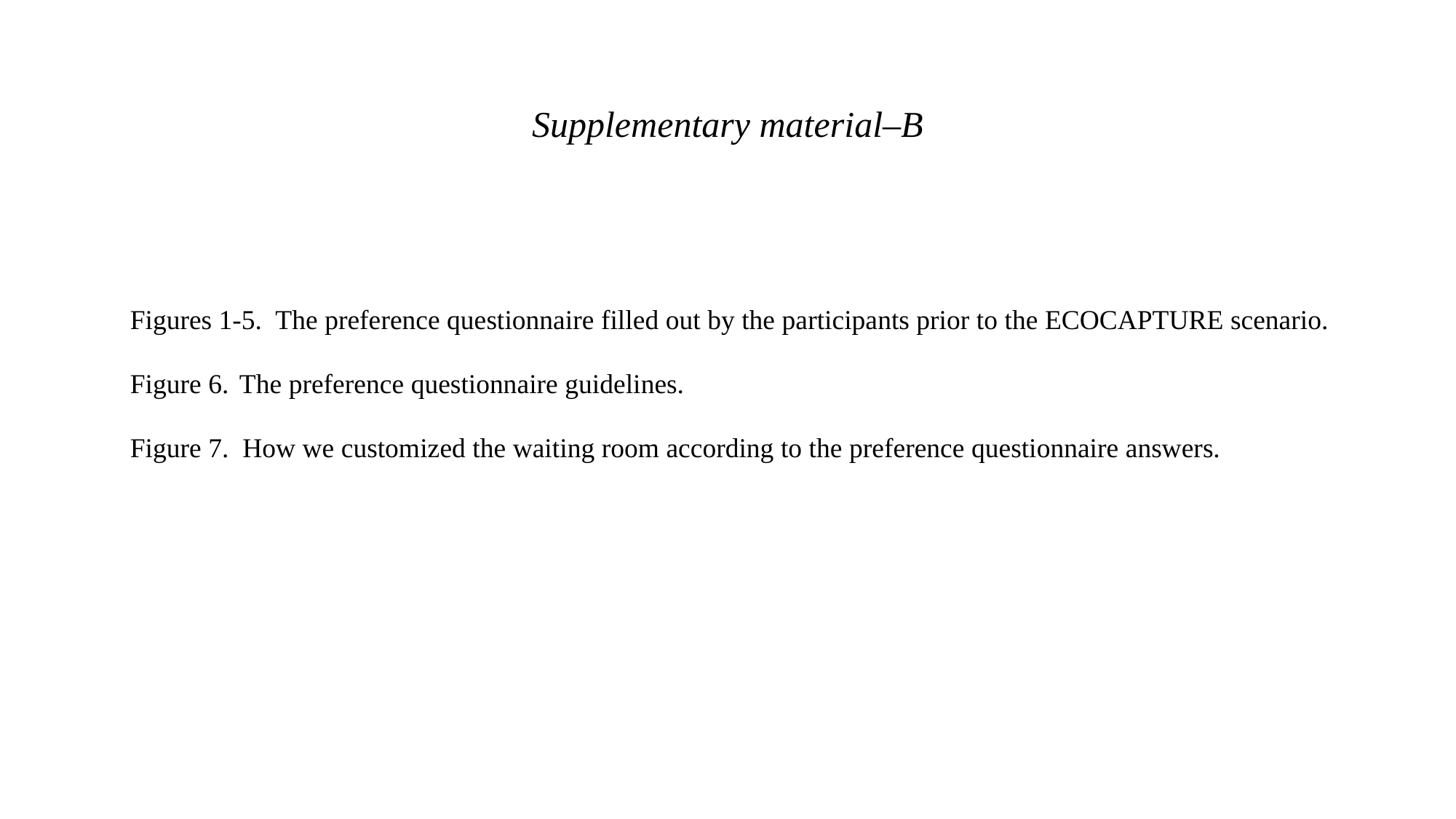

# Supplementary material–B
Figures 1-5. The preference questionnaire filled out by the participants prior to the ECOCAPTURE scenario.
Figure 6. 	The preference questionnaire guidelines.
Figure 7. How we customized the waiting room according to the preference questionnaire answers.

## Slide 2
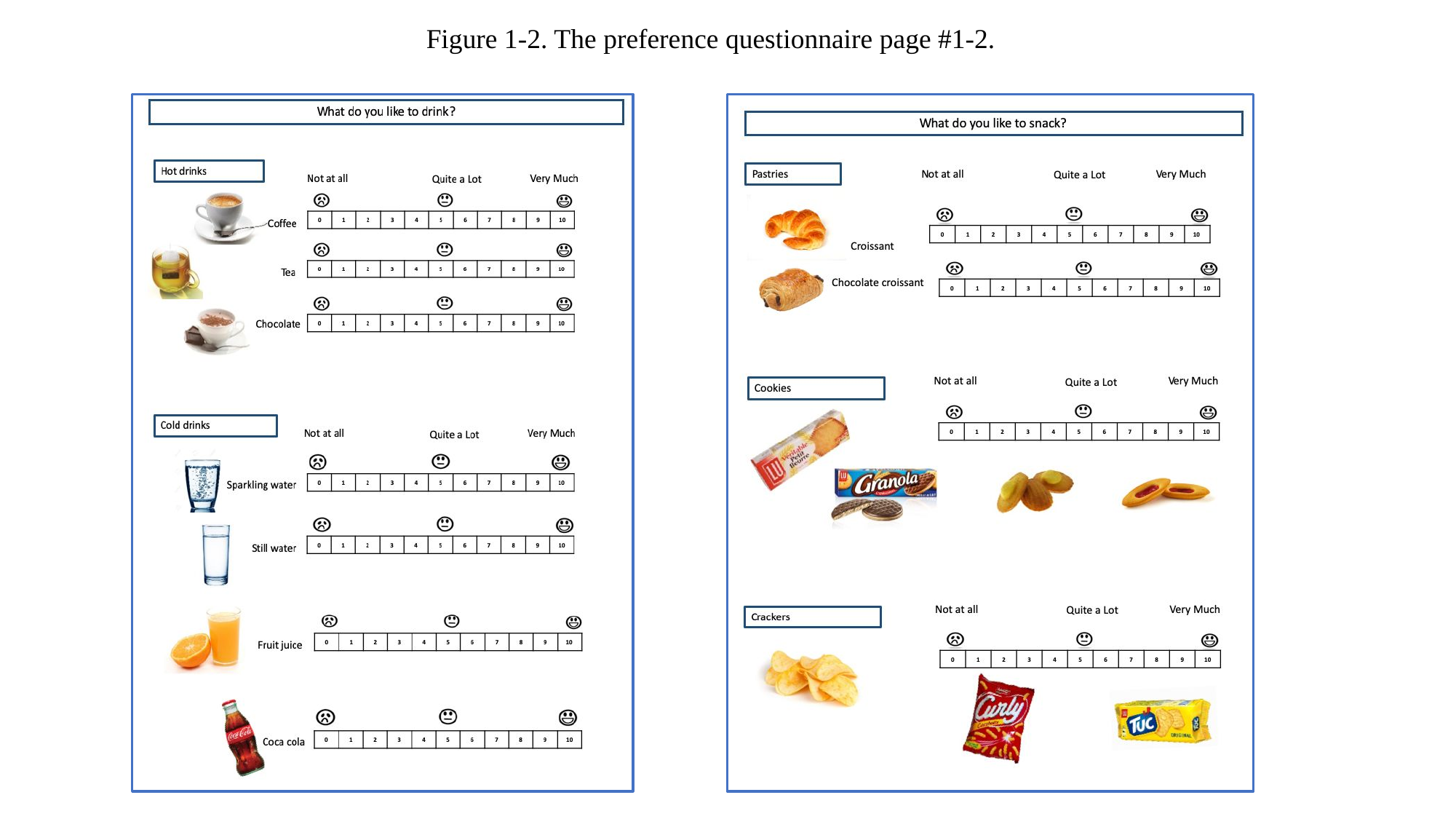

# Figure 1-2. The preference questionnaire page #1-2.

## Slide 3
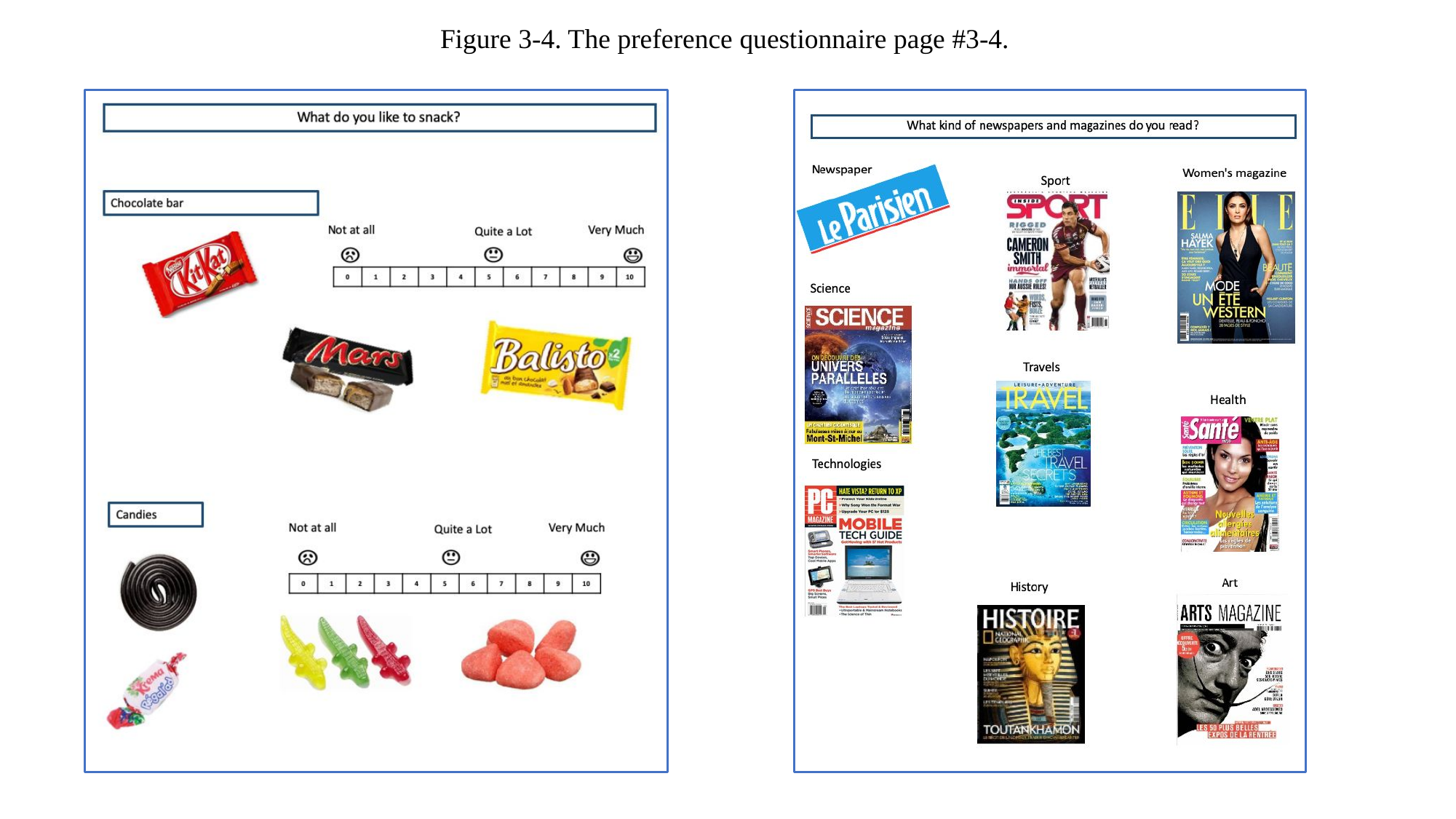

# Figure 3-4. The preference questionnaire page #3-4.

## Slide 4
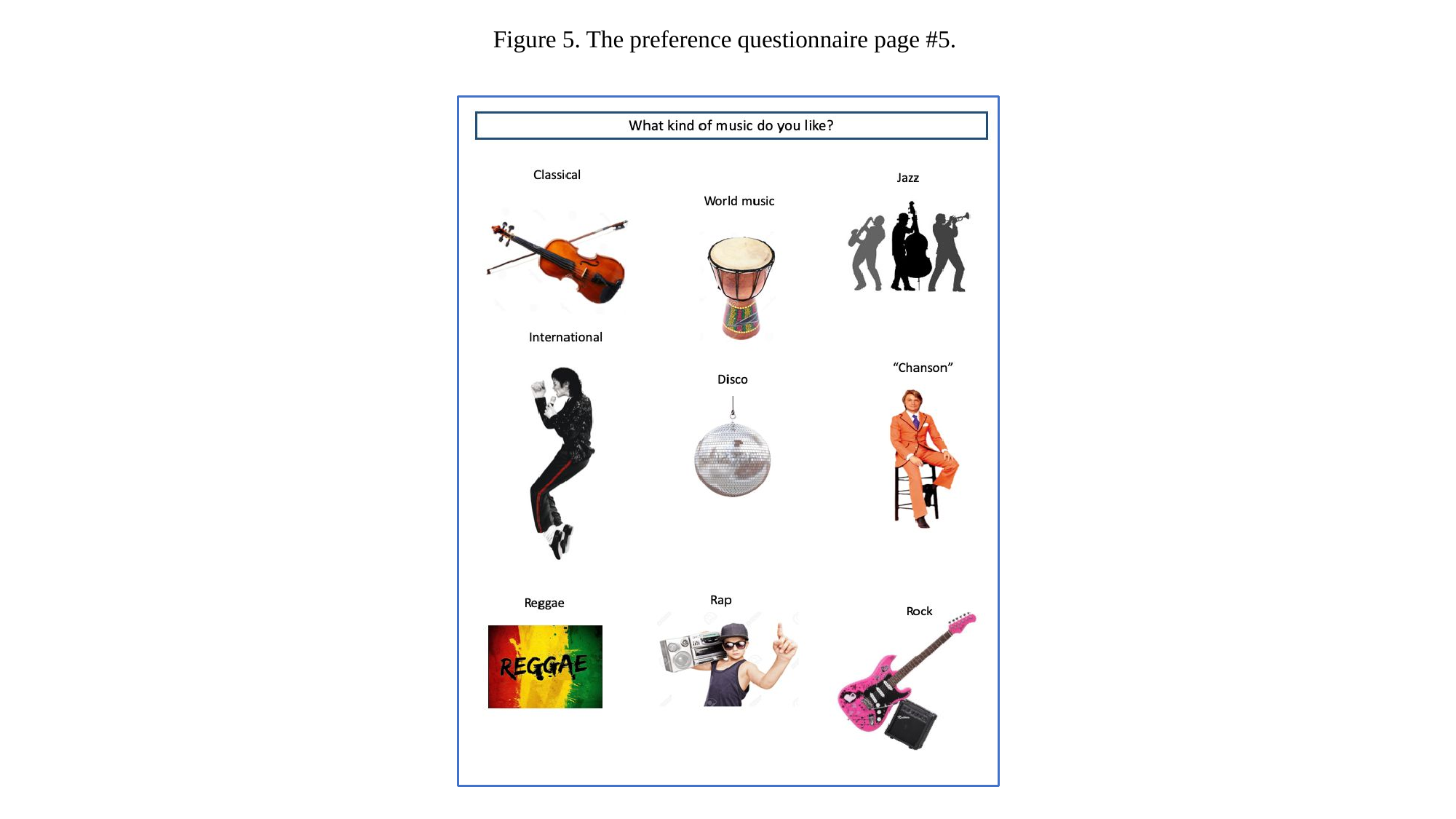

# Figure 5. The preference questionnaire page #5.

## Slide 5
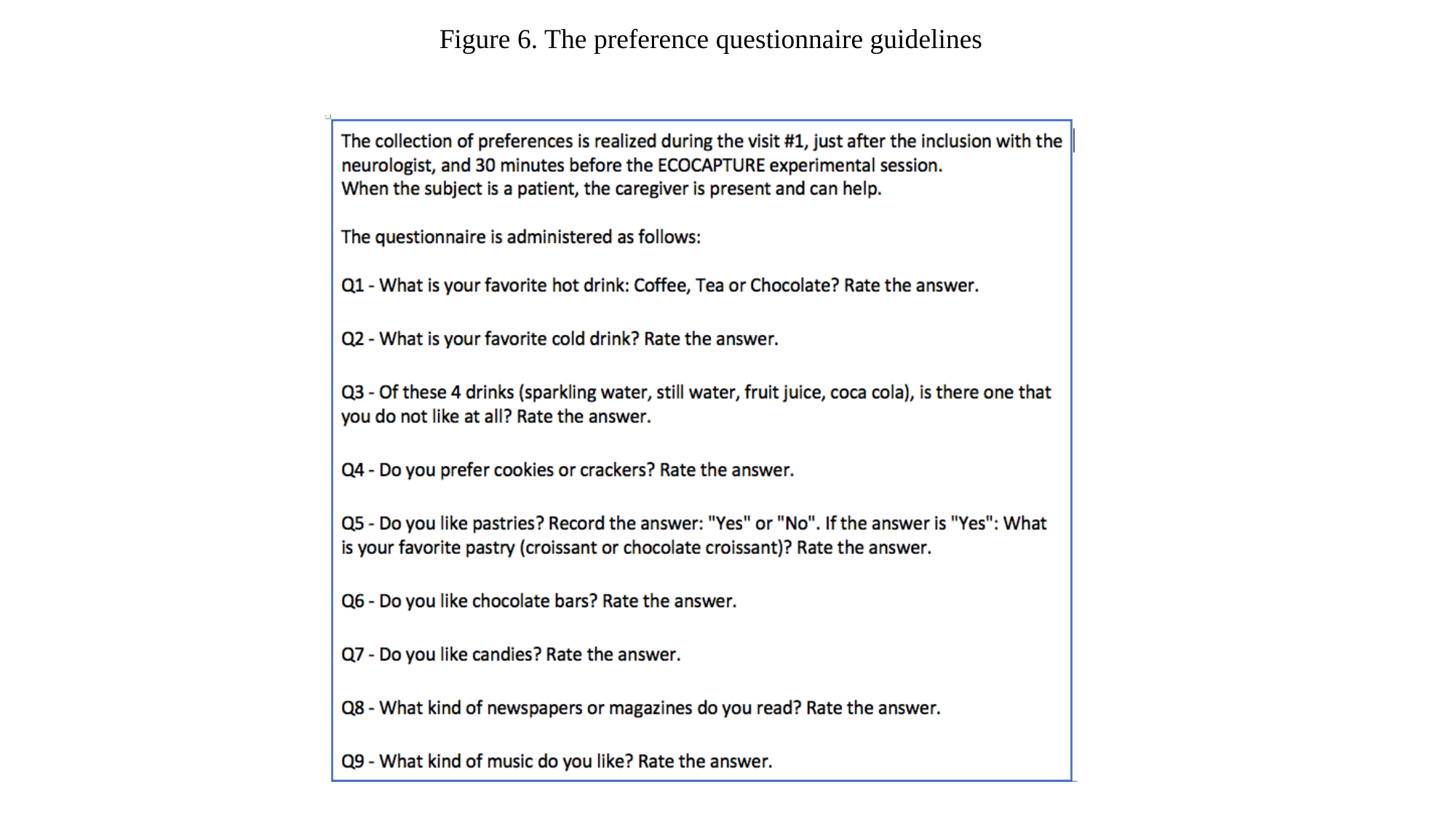

# Figure 6. The preference questionnaire guidelines

## Slide 6
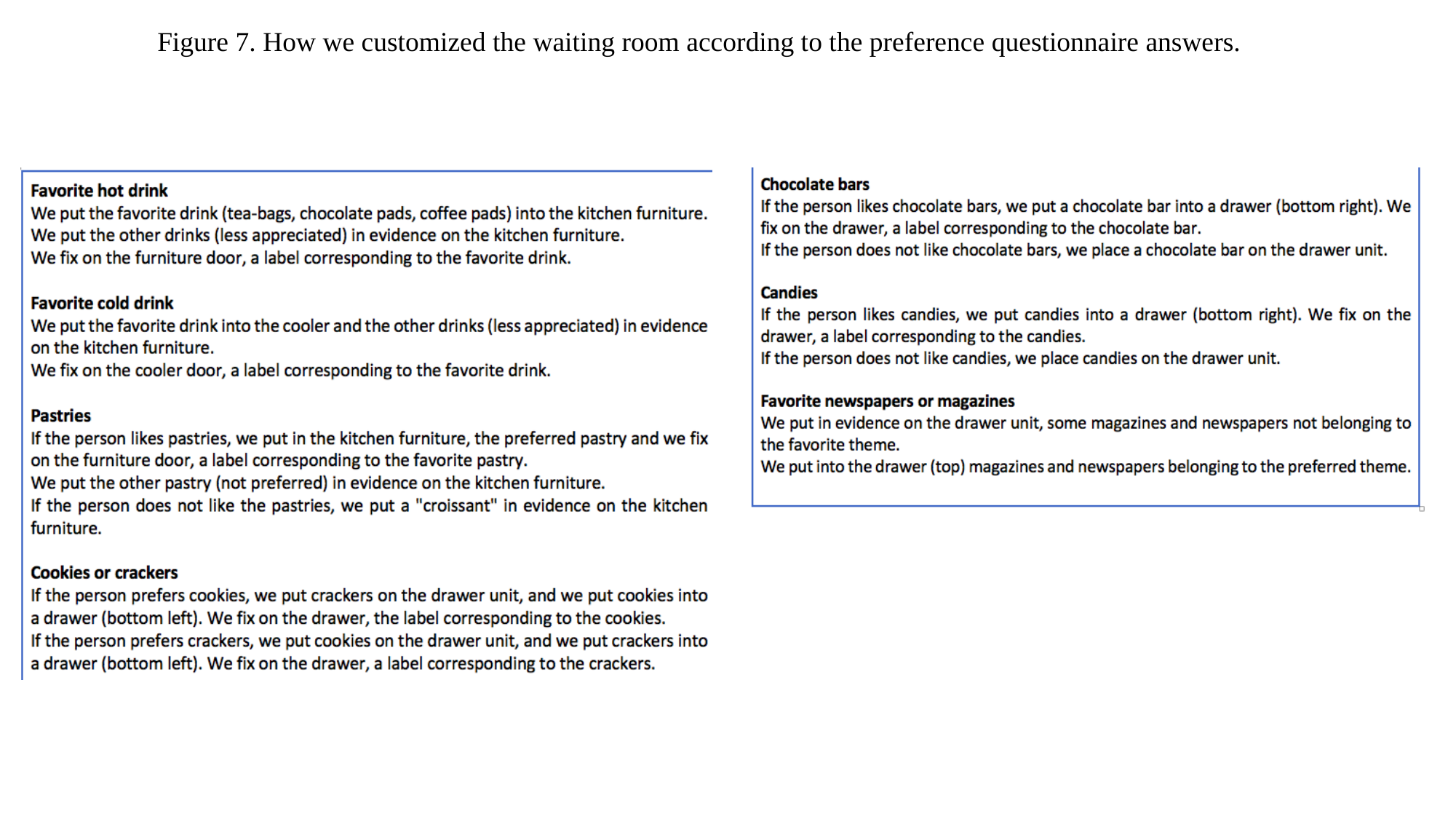

# Figure 7. How we customized the waiting room according to the preference questionnaire answers.
