# Supplementary material for: Exploration Deficits Under Ecological Conditions as a Marker of Apathy in Frontotemporal Dementia
Source: Front Neurol. 2019 Aug 28;10:941. doi: 10.3389/fneur.2019.00941 (PMC6736613; doi:10.3389/fneur.2019.00941)
Supplement: Supplementary file 3 [file Presentation_3.PPTX]

## Slide 1
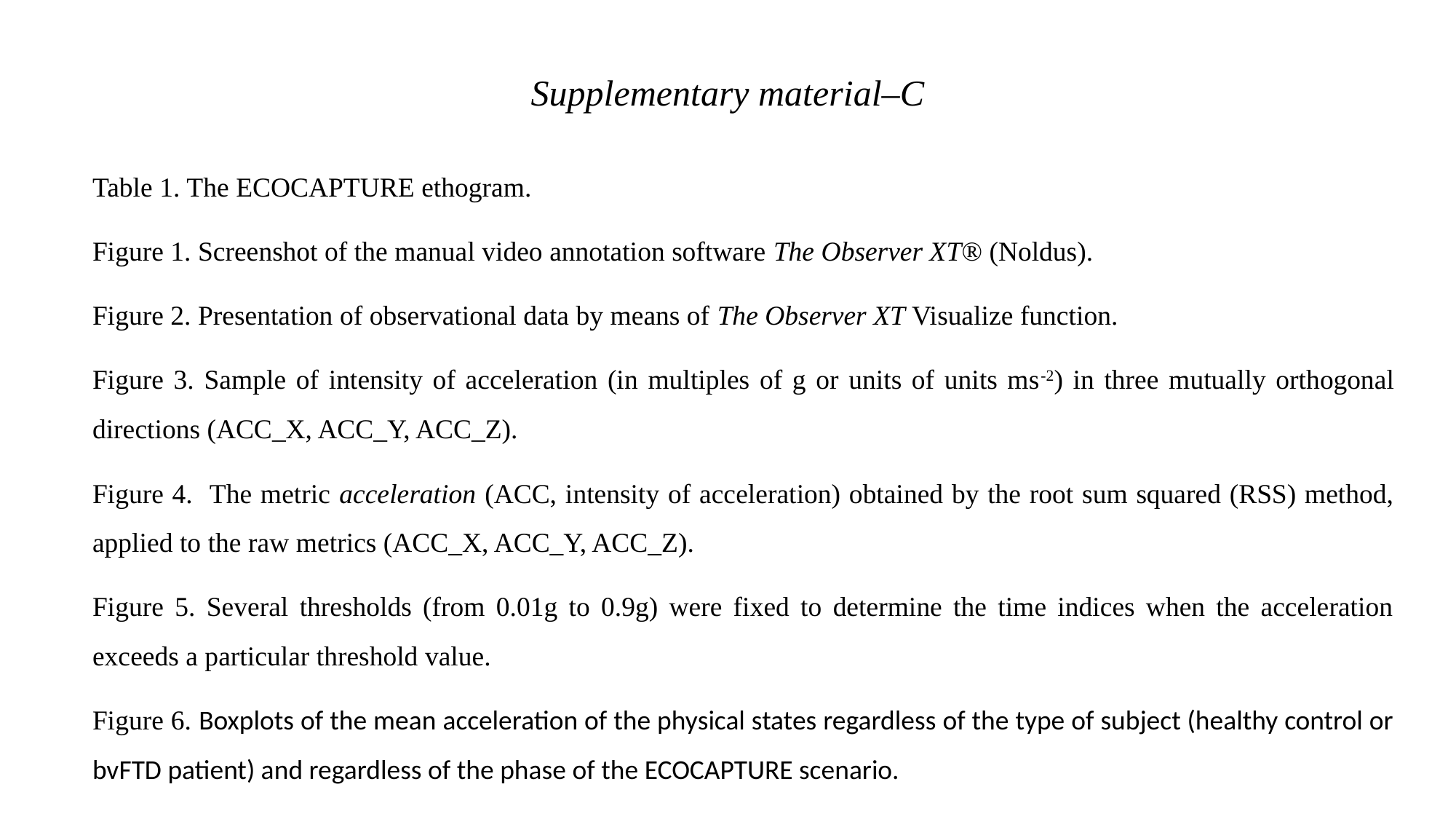

# Supplementary material–C
Table 1. The ECOCAPTURE ethogram.
Figure 1. Screenshot of the manual video annotation software The Observer XT® (Noldus).
Figure 2. Presentation of observational data by means of The Observer XT Visualize function.
Figure 3. Sample of intensity of acceleration (in multiples of g or units of units ms-2) in three mutually orthogonal directions (ACC_X, ACC_Y, ACC_Z).
Figure 4. The metric acceleration (ACC, intensity of acceleration) obtained by the root sum squared (RSS) method, applied to the raw metrics (ACC_X, ACC_Y, ACC_Z).
Figure 5. Several thresholds (from 0.01g to 0.9g) were fixed to determine the time indices when the acceleration exceeds a particular threshold value.
Figure 6. Boxplots of the mean acceleration of the physical states regardless of the type of subject (healthy control or bvFTD patient) and regardless of the phase of the ECOCAPTURE scenario.

## Slide 2
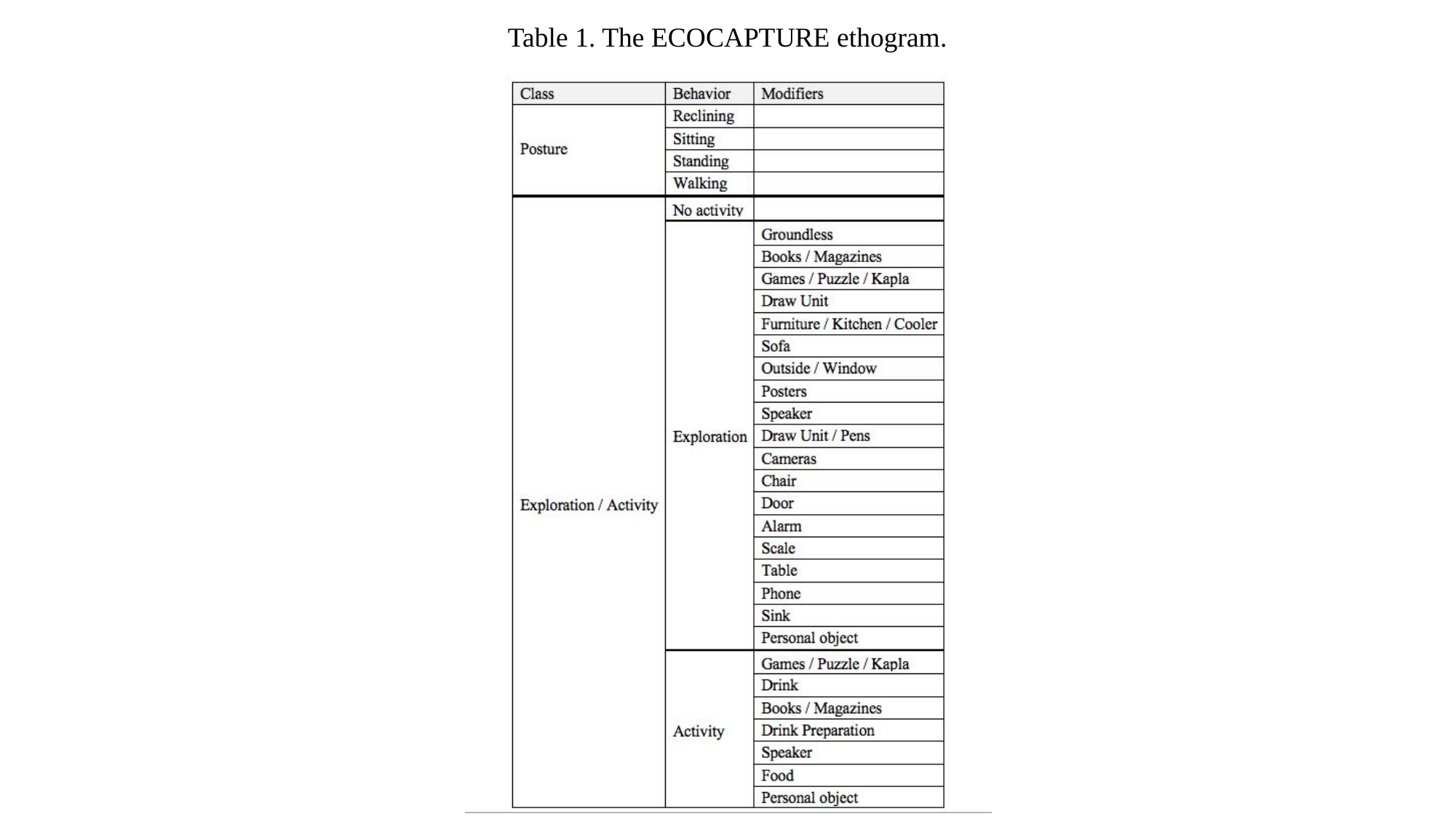

# Table 1. The ECOCAPTURE ethogram.

## Slide 3
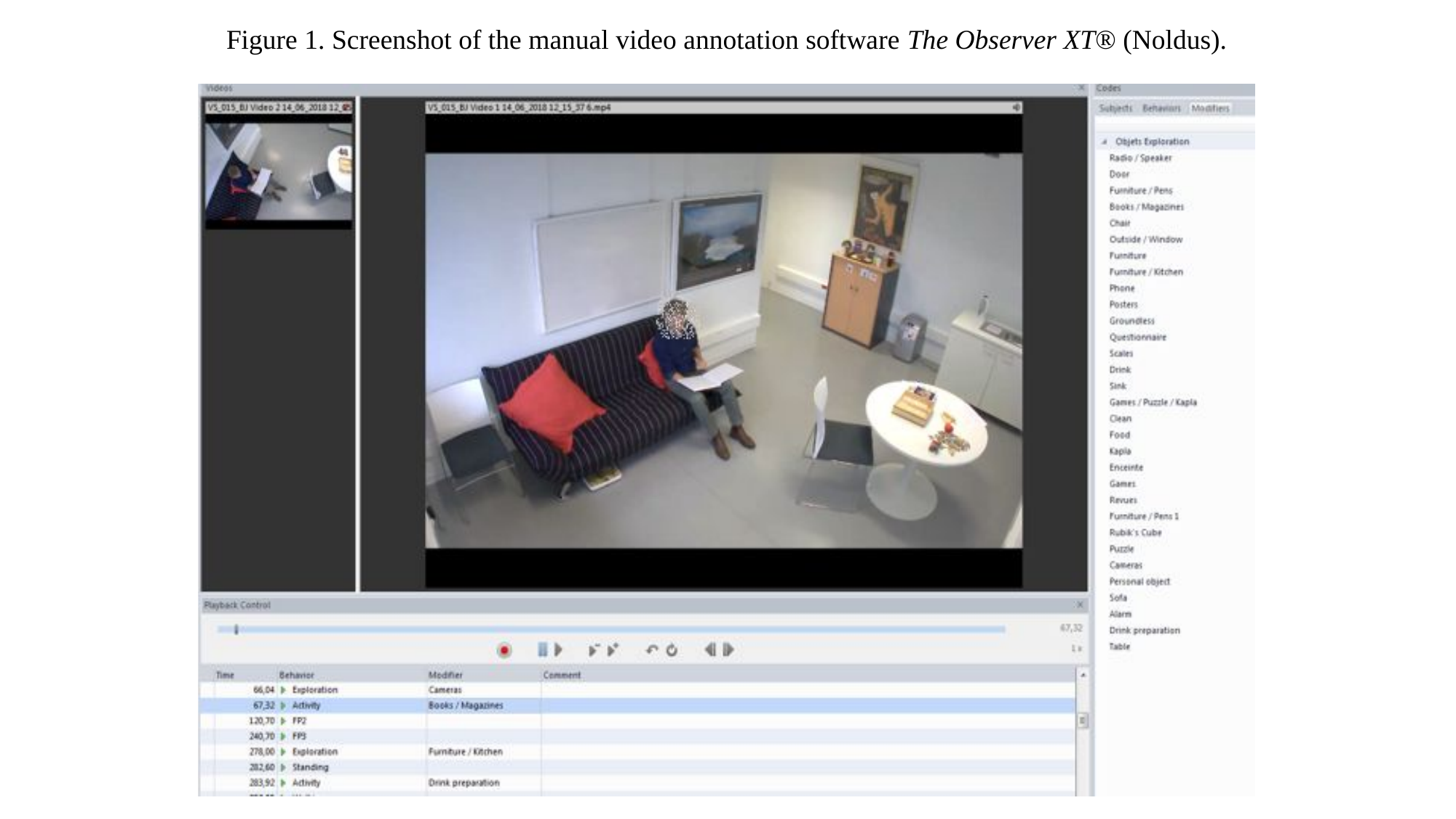

# Figure 1. Screenshot of the manual video annotation software The Observer XT® (Noldus).

## Slide 4
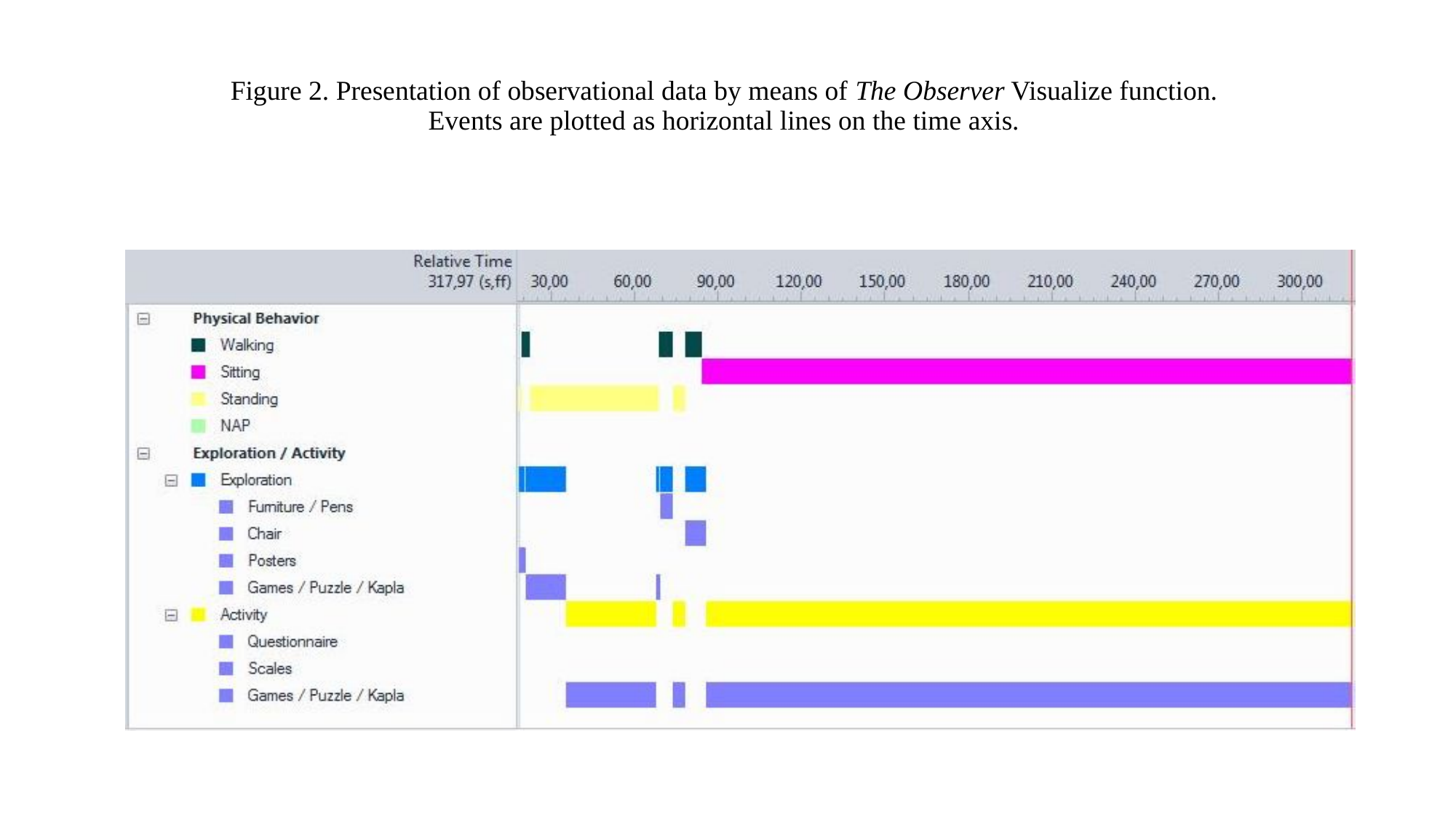

# Figure 2. Presentation of observational data by means of The Observer Visualize function. Events are plotted as horizontal lines on the time axis.

## Slide 5
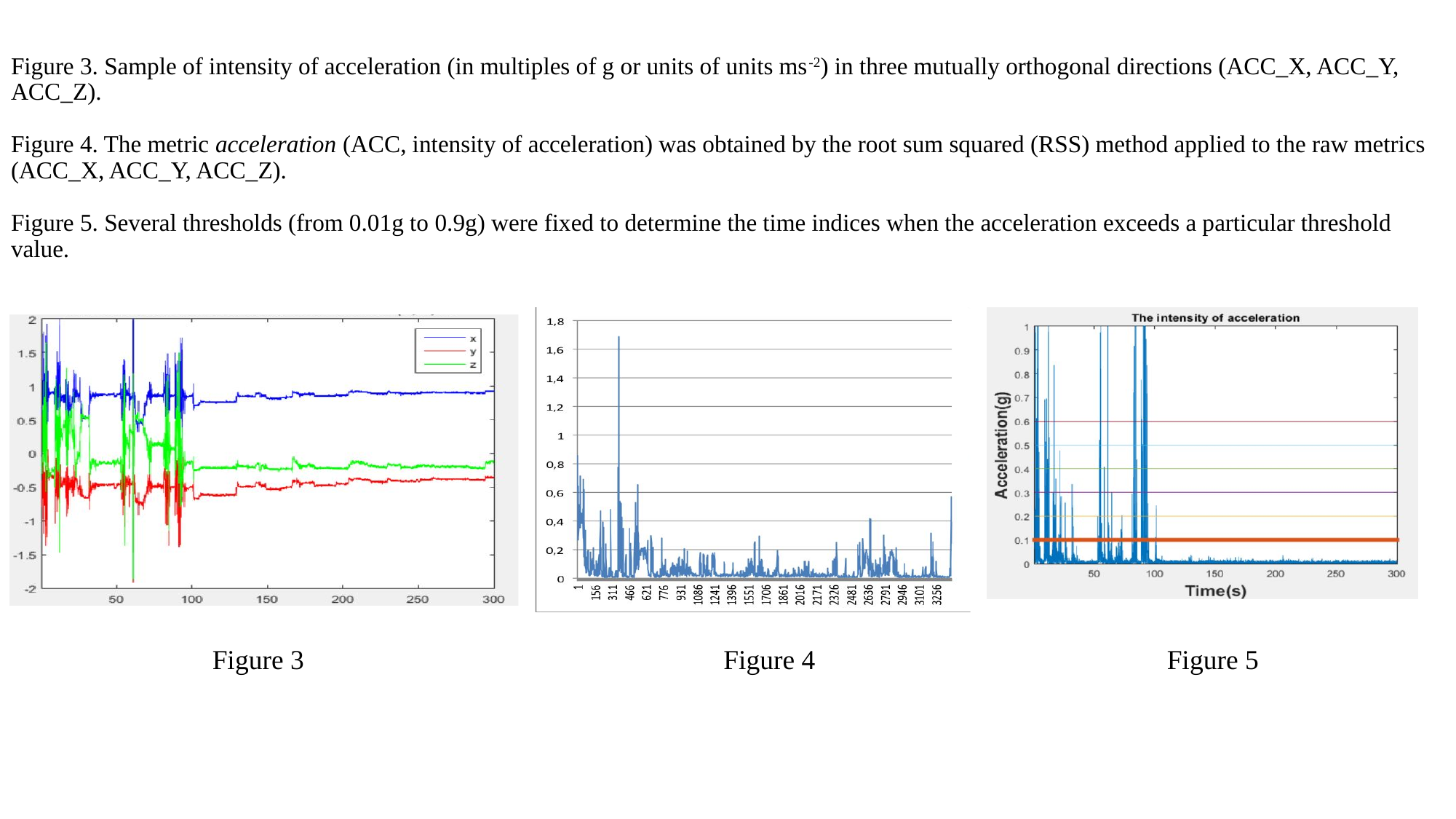

# Figure 3. Sample of intensity of acceleration (in multiples of g or units of units ms-2) in three mutually orthogonal directions (ACC_X, ACC_Y, ACC_Z). Figure 4. The metric acceleration (ACC, intensity of acceleration) was obtained by the root sum squared (RSS) method applied to the raw metrics (ACC_X, ACC_Y, ACC_Z). Figure 5. Several thresholds (from 0.01g to 0.9g) were fixed to determine the time indices when the acceleration exceeds a particular threshold value.
	 Figure 3				 Figure 4			 Figure 5

## Slide 6
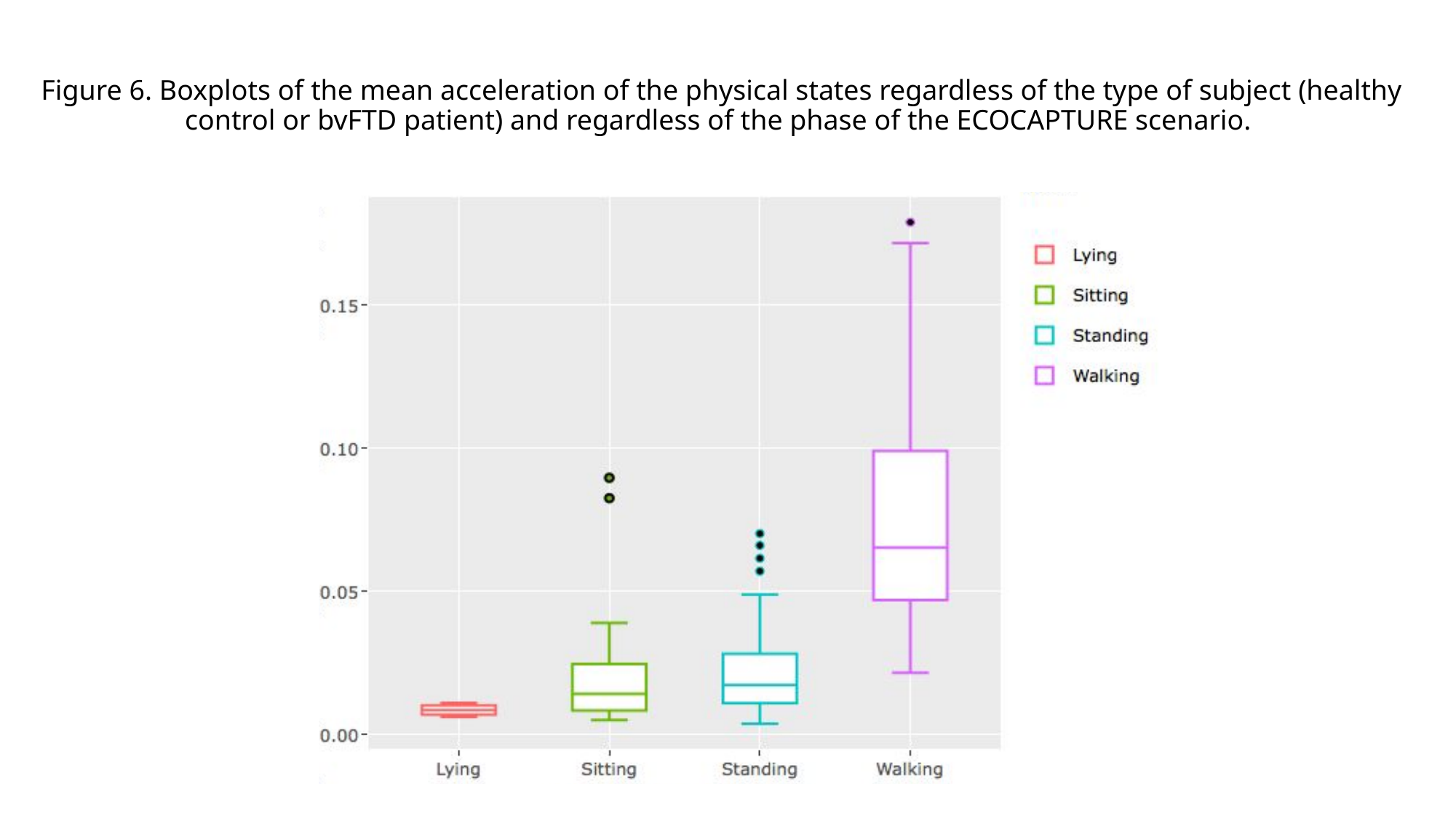

# Figure 6. Boxplots of the mean acceleration of the physical states regardless of the type of subject (healthy control or bvFTD patient) and regardless of the phase of the ECOCAPTURE scenario.
